# Declaring and Diagnosing Research Designs

**DOI:** 10.1017/s0003055419000194

**Published:** 2019-05-31

**Authors:** GRAEME BLAIR, JASPER COOPER, ALEXANDER COPPOCK, MACARTAN HUMPHREYS

**Affiliations:** University of California, Los Angeles; University of California, San Diego; Yale University; WZB Berlin and Columbia University

## Abstract

*Researchers need to select high-quality research designs and communicate those designs clearly to readers. Both tasks are difficult. We provide a framework for formally “declaring” the analytically relevant features of a research design in a demonstrably complete manner, with applications to qualitative, quantitative, and mixed methods research. The approach to design declaration we describe requires defining a model of the world (*M*), an inquiry (*I*), adatastrategy(*D*), andananswerstrategy(*A*). Declaration of these features in code provides sufficient information for researchers and readers to use Monte Carlo techniques to diagnose properties such as power, bias, accuracy of qualitative causal inferences, and other “diagnosands.” Ex ante declarations can be used to improve designs and facilitate preregistration, analysis, and reconciliation of intended and actual analyses. Ex post declarations are useful for describing, sharing, reanalyzing, and critiquing existing designs. We provide open-source software, DeclareDesign, to implement the proposed approach.*

As empirical social scientists, we routinely face two research design problems. First, we need to select high-quality designs, given resource constraints. Second, we need to communicate those designs to readers and reviewers.

To select strong designs, we often rely on rules of thumb, simple power calculators, or principles from the methodological literature that typically address one component of a design while assuming optimal conditions for others. These relatively informal practices can result in the selection of suboptimal designs, or worse, designs that are simply too weak to deliver useful answers.

To convince others of the quality of our designs, we often defend them with references to previous studies that used similar approaches, with power analyses that may rely on assumptions unknown even to ourselves, or with *ad hoc* simulation code. In cases of dispute over the merits of different approaches, disagreements sometimes fall back on first principles or epistemological debates rather than on demonstrations of the conditions under which one approach does better than another.

In this paper we describe an approach to address these problems. We introduce a framework—MIDA—that asks researchers to specify information about their background model (*M*), their inquiry (*I*), their data strategy (*D*), and their answer strategy (*A*). We then introduce the notion of “diagnosands,” or quantitative summaries of design properties. Familiar diagnosands include statistical power, the bias of an estimator with respect to an estimand, or the coverage probability of a procedure for generating confidence intervals. We say a design declaration is “diagnosand-complete” when a diagnosand can be estimated from the declaration. We do not have a general notion of a complete design, but rather adopt an approach in which the purposes of the design determine which diagnosands are valuable and in turn what features must be declared. In practice, domain-specific standards might be agreed upon among members of particular research communities. For instance, researchers concerned about the policy impact of a given treatment might require a design that is diagnosand-complete for an out-of-sample diag- nosand, such as bias relative to the population average treatment effect. They may also consider a diagnosand directly related to policy choices, such as the probability of making the right policy decision after research is conducted.

We acknowledge that although many aspects of design quality can be assessed through design diagnosis, many cannot. For instance the contribution to an academic literature, relevance to a policy decision, and impact on public debate are unlikely to be quantifiable ex ante.

Using this framework, researchers can *declare* a research design as a computer code object and then *diagnose* its statistical properties on the basis of this declaration. We emphasize that the term “declare” does not imply a public declaration or even necessarily a declaration before research takes place. A researcher may declare the features of designs in our framework for their own understanding and declaring designs may be useful before or after the research is implemented. Researchers can declare and diagnose their designs with the companion software for this paper, DeclareDesign, but the principles of design declaration and diagnosis do not depend on any particular software implementation.

The formal characterization and diagnosis of designs before implementation can serve many purposes. First, researchers can learn about and improve their inferential strategies. Done at this stage, diagnosis of a design and alternatives can help a researcher select from a range of designs, conditional upon beliefs about the world. Later, a researcher may include design declaration and diagnosis as part of a preanalysis plan or in a funding request. At this stage, the full specification of a design serves a communication function and enables third parties to understand a design and an author’s intentions. Even if declared ex-post, formal declaration still has benefits. The complete characterization can help readers understand the properties of a research project, facilitate transparent replication, and can help guide future (re-)analysis of the study data.

The approach we describe is clearly more easily applied to some types of research than others. In prospective confirmatory work, for example, researchers may have access to all design-relevant information prior to launching their study. For more inductive research, by contrast, researchers may simply not have enough information about possible quantities of interest to declare a design in advance. Although in some cases the design may still be usefully declared *ex post,* in others it may not be possible to fully reconstruct the inferential procedure after the fact. For instance, although researchers might be able to provide compelling grounds for their inferences, they may not be able to describe what inferences they would have drawn had different data been realized. This may be particularly true of interpretivist approaches and approaches to process tracing that work backwards from outcomes to a set of possible causes that cannot be prespecified. We acknowledge from the outset that variation in research strategy limits the utility of our procedure for different types of research. Even still, we show that our framework can accommodate discovery, qualitative inference, and different approaches to mixed methods research, as well as designs that focus on “effects-of-causes” questions, often associated with quantitative approaches, and “causes-of-effects” questions, often associated with qualitative approaches.

Formally declaring research designs as objects in the manner we describe here brings, we hope, four benefits. It can facilitate the diagnosis of designs in terms of their ability to answer the questions we want answered under specified conditions; it can assist in the improvement of research designs through comparison with alternatives; it can enhance research transparency by making design choices explicit; and it can provide strategies to assist principled replication and reanalysis of published research.

## RESEARCH DESIGNS AND DIAGNOSANDS

We present a general description of a research design as the specification of a problem and a strategy to answer it. We build on two influential research design frameworks. King, Keohane, and Verba (1994, 13) enumerate four components of a research design: a theory, a research question, data, and an approach to using the data. Geddes (2003) articulates the links between theory formation, research question formulation, case selection and coding strategies, and strategies for case comparison and inference. In both cases, the set of components are closely aligned to those in the framework we propose. In our exposition, we also employ elements from Pearl’s (2009) approach to structural modeling, which provides a syntax for mapping design inputs to design outputs as well as the potential outcomes framework as presented, for example, in Imbens and Rubin (2015), which many social scientists use to clarify their inferential targets. We characterize the design problem at a high level of generality with the central focus being on the relationship between questions and answer strategies. We further situate the framework within existing literature below.

### Elements of a Research Design

The specification of a problem requires a description of the world and the question to be asked about the world as described. Providing an answer requires a description of what information is used and how conclusions are reached given this information.

At its most basic we think of a research design as including four elements 〈*M, I, D, A*〉:

1. A causal **model**, M, of how the world works.^1^ In general, following Pearl’s definition of a probabilistic causal model (Pearl 2009) we assume that a model contains three core elements. First, a specification of the variables *X* about which research is being conducted. This includes endogenous and exogenous variables (*V* and *U* respectively) and the ranges of these variables. In the formal literature this is sometimes called the *signature* of a model (e.g., Halpern 2000). Second, a specification of how each endogenous variable depends on other variables (the “functional relations” or, as in Imbens and Rubin (2015), “potential outcomes”), *F.* Third, a probability distribution over exogenous variables, *P(U).*
2. An **inquiry**, *I*, about the distribution of variables, *X*, perhaps given interventions on some variables. Using Pearl’s notation we can distinguish between questions that ask about the conditional values of variables, such as Pr(*X*_1_|*X*_2_ = 1) and questions that ask about values that would arise under interventions: Pr(*X*_1_|*do*(*X*_2_ = 1))^2^. We let *a*^*M*^ denote the answer to *I under the model.* Conditional on the model, *a*^*M*^ is the value of the estimand, the quantity that the researcher wants to learn about.
3. A **data** strategy, *D*, generates data *d* on *X* under model *M* with probability *P*_*M*_(*d*|*D*). The data strategy includes sampling strategies and assignment strategies, which we denote with *P*_*S*_ and *P*_*Z*_ respectively. Measurement techniques are also a part of data strategies and can be thought of as procedures by which unobserved latent variables are mapped (possibly with error) into observed variables.
4. An **answer** strategy, *A*, that generates answer *a*^*A*^ using data *d.*

A key feature of this bare specification is that if *M, D,* and *A* are sufficiently well described, the answer to question *I* has a distribution *P*_*M*_*(a*^*A*^*|D).* Moreover, one can construct a distribution of comparisons of this answer to the correct answer, under *M*, for example by assessing *P*_*M*_(*a*^*A*^ − *a*^*M*^*|D*). One can also compare this to results under different data or analysis strategies, $P_{M}\left(a^{A}-a^{M}|D^{\prime }\right)$ and $P_{M}\left(a^{A^{\prime }}-a^{M}|D\right)$, and to answers generated under alternative models, $P_{M}\left(a^{A}-a^{M^{\prime }}|D\right)$, as long as these possess signatures that are consistent with inquiries and answer strategies.

MIDA captures the analysis-relevant features of a design, but it does not describe substantive elements, such as how theories are derived, how interventions are implemented, or even, qualitatively, how outcomes are measured. Yet many other aspects of a design that are not explicitly labeled in these features enter into this framework if they are analytically relevant. For example, if treatment effects decay, logistical details of data collection (such as the duration of time between a treatment being administered and endline data collection) may enter into the model. Similarly, if a researcher anticipates noncompliance, substantive knowledge of how treatments are taken up can be included in many parts of the design.

### Diagnosands

The ability to calculate distributions of answers, given a model, opens multiple avenues for assessment and critique. How good is the answer you expect to get from a given strategy? Would you do better, given some desideratum, with a different data strategy? With a different analysis strategy? How good is the strategy if the model is wrong in one way or another?

To allow for this kind of *diagnosis* of a design, we introduce two further concepts, both functions of research designs. These are quantities that a researcher or a third party could calculate with respect to a design.

1. A **diagnostic statistic** is a summary statistic generated from a “run” of a design—that is, the results given a possible realization of variables, given the model and data strategy. For example the statistic: *e* = “difference between the estimated and the actual average treatment effect” is a diagnostic statistic that requires specifying an estimand. The statistic s=1(p≤0.05), interpreted as “the result is considered statistically significant at the 5% level,” is a diagnostic statistic that does not require specifying an estimand, but it does presuppose an answer strategy that reports a *p*-value.

Diagnostic statistics are governed by probability distributions that arise because both the model and the data generation, given the model, may be stochastic.

- A **diagnosand** is a summary of the distribution of a diagnostic statistic. For example, (expected) *bias* in the estimated treatment effect is E(e) and statistical *power* is E(s).

To illustrate, consider the following design. A model *M* specifies three variables *X*, *Y*, and *Z* defined on the real number line that form the signature. In additional we assume functional relationships between them that allow for the possibility of confounding (for example, *Y = bX + Z +* ε_*Y*_*; X = Z +* ε_*X*_, with Z, ε_*X*_, ε_*Z*_ distributed standard normal). The inquiry *I* is “what would be the average effect of a unit increase in *X* on *Y* in the population?” The specification of this question depends on the signature of the model, but not the functional relations of the model. The answer provided by the model does of course depend on the functional relations. Consider now a data strategy, *D*, in which data are gathered on *X* and *Y* for *n* randomly selected units. An answer *a*^*A*^, is then generated using ordinary least squares as the answer strategy, *A*.

We have specified all the components of MIDA. We now ask: How strong is this research design? One way to answer this question is with respect to the diagnosand “bias.” Here the model provides an answer, *a*^*M*^, to the inquiry, so the distribution of bias *given the model, a*^*A*^ ***–*** *a*^*M*^, can be calculated.

In this example, the expected performance of the design may be poor, as measured by the bias diag- nosand, because the data and analysis strategy do not handle the confounding described by the model (see Supplementary Materials Section 1 for a formal declaration and diagnosis of this design). In comparison, better performance may be achieved through an alternative data strategy (e.g., where *D′* randomly assigned *X* before recording *X* and *Y*) or an alternative analysis strategy (e.g., *A′* conditions on Z). These design evaluations depend on the model, and so one might reasonably ask how performance would look were the model different (for example, if the underlying process involved nonlinearities).

In all cases, the evaluation of a design depends on the assessment of a diagnosand, and comparing the diagnoses to what could be achieved under alternative designs.

### Choice of Diagnosands

What diagnosands should researchers choose? Although researchers commonly focus on statistical power, a larger range of diagnosands can be examined and may provide more informative diagnoses of design quality. We list and describe some of these in Table 1, indicating for each the design information that is required in order to calculate them.

The set listed here includes many canonical diagnosands used in classical quantitative analyses. Diagnosands can also be defined for design properties that are often discussed informally but rarely subjected to formal investigation. For example one might define an inference as “robust” if the same inference is made under different analysis strategies. One might conclude that an intervention gives “value for money” if estimates are of a certain size and be interested in the probability that a researcher in correct in concluding that an intervention provides value for money.

We believe there is not yet a consensus around diagnosands for qualitative designs. However, in certain treatments clear analogues of diagnosands exist, such as sampling bias or estimation bias (e.g., Herron and Quinn 2016). There are indeed notions of power, coverage, and consistency for QCA researchers (e.g., Baumgartner and Thiem 2017; Rohlfing 2018) and concerns around correct identification of causes of effects, or of causal pathways, for scholars using process-tracing (e.g., Bennett 2015; Fairfield and Charman 2017; Humphreys and Jacobs 2015; Mahoney 2012).

Though many of these diagnosands are familiar to scholars using frequentist approaches, analogous diagnosands can be used to assess Bayesian estimation strategies (see Rubin 1984), and as we illustrate below, some diagnosands are unique to Bayesian answer strategies.

Given that there are many possible diagnosands, the overall evaluation of a design is both multi-dimensional and qualitative. For some diagnosands, quality thresholds have been established through common practice, such as the standard power target of 0.80. Some researchers are unsatisfied unless the “bias” diagnosand is exactly zero. Yet for most diagnosands, we only have a sense of better and worse, and improving one can mean hurting another, as in the classic bias-variance tradeoff. Our goal is not to dichotomize designs into high and low quality, but instead to facilitate the assessment of design quality on dimensions important to researchers.

### What is a Complete Research Design Declaration?

A declaration of a research design that is in some sense complete is required in order to implement it, communicate its essential features, and to assess its properties. Yet existing definitions make clear that there is no single conception of a complete research design: at the time of writing, the Consolidated Standards of Reporting Trials (CONSORT) Statement widely used in medicine includes 22 features, while other proposals range from nine to 60 components^3^.

We propose a conditional conception of completeness: we say a design is “diagnosand-complete” for a given diagnosand if that diagnosand can be calculated from the declared design. Thus a design that is diagnosand-complete for one diagnosand may not be for another. Consider, for example, the diagnosand statistical power. Power is the probability of obtaining a statistically significant result. Equivalently, it is the probability that the *p*-value is lower than a critical value (e.g., 0.005). Thus, power-completeness requires that the answer strategy return a *p*-value and a significance threshold be specified. It does not, however, require a well-defined estimand, such as a true effect size (see Table 1 where, for a power diagnosand, there is no check under I). In contrast, bias- or RMSE-completeness does not require a hypothesis test, but does require the specification of an estimand.

Diagnosand-completeness is a desirable property to the extent that it means a diagnosand can be calculated. How useful diagnosand-completeness is depends on whether the diagnosand is worth knowing. Thus, evaluating completeness should focus first on whether diagnosands for which completeness holds are indeed useful ones.

The utility of a diagnosis depends in part on whether the information underlying declaration is believable. For instance, a design may be bias-complete, but only under the assumptions of a given spillover structure. Readers might disagree with these assumptions. Even in this case, however, an advantage of declaration is a clarification of the conditions for completeness.

## EXISTING APPROACHES TO LEARNING ABOUT RESEARCH DESIGNS

Much quantitative research design advice focuses on one aspect of design at a time, rather than on the ways in which multiple components of a research design relate to each other. Statistics articles and textbooks tend to focus on a specific class of estimators (Angrist and Pischke 2008; Imbens and Rubin 2015; Rosenbaum 2002), set of estimands (Heckman, Urzua, and Vytlacil 2006; Imai, King, and Stuart 2008; Deaton 2010; Imbens 2010), data collection strategies (Lohr 2010), or ways of thinking about data-generation models (Gelman and Hill 2006; Pearl 2009). In Shadish, Cook, and Campbell (2002, 156), for example, the “elements of a design” consist of “assignment, measurement, comparison groups and treatments,” a definition that does not include questions of interest or estimation strategies. In some instances, quantitative researchers do present multiple elements of research design. Gerber and Green (2012), for example, examine data-generating models, estimands, assignment and sampling strategies, and estimators for use in experimental causal inference; and Shadish, Cook, and Campbell (2002) and Dunning (2012) similarly describe the various aspects of designing quasi-experimental research and exploiting natural experiments.

In contrast, a number of qualitative treatments focus on integrating the many stages of a research design, from theory generation, to case selection, measurement, and inference. In an influential book on mixed method research design for comparative politics, for example, Geddes (2003) articulates the links between theory formation (M), research question formulation (I), case selection and coding strategies (D), and strategies for case comparison and inference (A). King, Keohane, and Verba (1994) and the ensuing discussion in Brady and Collier (2010) highlight how alternative qualitative strategies present tradeoffs in terms of diagnosands such as bias and generalizability. However, few of these texts investigate those diagnosands formally in order to measure the size of the tradeoffs between alternative qualitative strategies^4^. Qualitative approaches, including process tracing and qualitative comparative analysis, sometimes appear almost hermetic, complete with specific epistemologies, types of research questions, modes of data gathering, and analysis. Though integrated, these strategies are often not formalized. And if they are, it is seldom in a way that enables comparison with other approaches or quantification of design tradeoffs.

MIDA represents an attempt to thread the needle between these two traditions. Quantifying the strength of designs necessitates a language for formally describing the essential features of a design. The relatively fragmented manner in which the quantitative design is thought of in existing work may produce real research risks for individual research projects. In contrast, the more holistic approaches of some qualitative traditions offer many benefits, but formal design diagnosis can be difficult. Our hope is that MIDA provides a framework for doing both at once.

A useful way to illustrate the fragmented nature of thinking on research design among quantitative scholars is to examine the tools that are actually used to do research design. Perhaps the most prominent of these are “power calculators.” These have an all-design flavor in the sense that they ask whether, given an answer strategy, a data collection strategy is likely to return a statistically significant result. Power calculations like these are done using formulae (e.g., Cohen 1977; Haseman 1978; Lenth 2001; Muller et al. 1992; Muller and Peterson 1984); software tools such as Web applications and general statistical software (e.g., easy power for R and Power and Sample Size for Stata) as well as standalone tools (e.g., Optimal Design, G*Power, nQuery, SPSS Sample Power); and sometimes Monte Carlo simulations.

In most cases these tools, though touching on multiple parts of a design, in fact leave almost no scope to describe what the data generating processes can be, what the questions of interest are, and what types of analyses will be undertaken. We conducted a census of currently available diagnostic tools (mainly power calculators) and assessed their ability to correctly diagnose three variants of a common experimental design, in which assignment probabilities are heterogeneous by block.^5^ The first variant simply uses a difference-in-means estimator (DIM), the second conditions on block fixed effects (BFE), and the third includes inverse- probability weighting to account for the heterogeneous assignment probabilities (BFE-IPW).

We found that the vast majority of tools used are unable to correctly characterize the tradeoffs these three variants present. As shown in Table 2, none of the tools was able to diagnose the design while taking account of important features that bias unweighted estimators.^6^ In our simulations the result is an overstatement of the power of the difference-in-means.

Because no tool was able to account for weighting in the estimator, none was able to calculate the power for the IPW-BFE answer strategy. Moreover, no tool sought to calculate the design’s bias, root mean- squared-error, or coverage (which require information on *I*). The companion software to this article, which was designed based on MIDA, illustrates that power is a misleading indicator of quality in this context. While the IPW-BFE estimator is better powered and less biased than the BFE estimator, its purported efficiency is misleading. IPW-BFE is better powered than DIM and BFE because it produces biased variance estimates that lead to a coverage probability that is too low. In terms of RMSE and the standard deviation of estimates, the IPW-BFE strategy does not outperform the BFE estimator. This exercise should not be taken as proof of the superiority of one strategy over another in general; instead we learn about their relative performance for particular diagnosands for the specific design declared.

We draw a number of conclusions from this review of tools.

First, researchers are generally not designing studies using the actual strategies that they will use to conduct analysis. From the perspective of the overall designs, the power calculations are providing the wrong answer.

Second, the tools can drive scholars toward relatively narrow design choices. The inputs to most power calculators are data strategy elements like the number of units or clusters. Power calculators do not generally focus on broader aspects of a design, like alternative assignment procedures or the choice of estimator. While researchers may have an awareness that such tradeoffs exist, quantifying the *extent* of the tradeoff is by no means obvious until the model, inquiry, data strategy, and answer strategy is declared in code.

Third, the tools focus attention on a relatively narrow set of questions for evaluating a design. While understanding power is important for some designs, the range of possible diagnosands of interest is much broader. Quantitative researchers tend to focus on power, when other diagnosands such as bias, coverage, or RMSE may also be important. MIDA makes clear, however, that these features of a design are often linked in ways that current practice obscures.

A second illustration of risks arising from a fragmented conceptualization of research design comes from debates over the disproportionate focus on estimators to the detriment of careful consideration of estimands. Huber (2013), for example, worries that the focus on identification leads researchers away from asking compelling questions. In the extreme, the estimators themselves (and not the researchers) appear to select the estimand of interest. Thus, Deaton (2010) highlights how instrumental variables approaches identify effects for a subpopulation of compliers. Who the compliers are is jointly determined by the characteristics of the subjects and also by the data strategy. The implied estimand (the Local Average Treatment Effect, sometimes called the Complier Average Causal Effect) may or may not be of theoretical interest. Indeed, as researchers swap one instrument for another, the implied estimand changes. Deaton’s worry is that researchers are getting an answer, but they do not know what the question is.^7^ Were the question posed as the average effect of a treatment, then the performance of the instrument would depend on how well the instrumental variables regression estimates that quantity, and not how well they answer the question for a different subpopulation. This is not done in usual practice, however, as estimands are often not included as part of a research design.

To illustrate risks arising from the combination of a fractured approach to design in the formal quantitative literature, and the holistic but often less formal approaches in the qualitative literature, we point to difficulties these approaches have in learning from each other.

Goertz and Mahoney (2012) tell a tale of two cultures in which qualitative and quantitative researchers differ not just in the analytic tools they use, but in very many ways, including, fundamentally, in their conceptualizations of causation and the kinds of questions they ask. The authors claim (though not all would agree) that qualitative researchers think of causation in terms of necessary and/or sufficient causes, whereas many quantitative researchers focus on potential outcomes, average effects, and structural equations. One might worry that such differences would preclude design declaration within a common framework, but they need not, at least for qualitative scholars that consider causes in counterfactual terms.^8^

For example, a representation of a causal process in terms of causal configurations might take the form: *Y = AB* + *C,* meaning that the presence of *A* and *B* or the presence of *C* is sufficient to produce *Y*. This configuration statement maps directly into a potential outcomes function (or structural equation) of the form *Y*(*A, B, C*) = max(*AB, C*). Given this, the marginal effect of one variable, conditional on others, can be translated to the conditions in which the variable is difference-making in the sense of altering relevant INUS^9^ conditions: *E(Y(A =* 1|*B*, *C)* − *Y(A =* 0|*B*, *C*)) = *E(B =* 1, *C =* 0).^10^ Describing these differences in notation as differences in notions of causality suggests that there is limited scope for considering designs that mix approaches, and that there is little that practitioners of one approach can say to practitioners of another approach. In contrast, clarification that the difference is one regarding the inquiry—i.e., which combinations of variables guarantee a given outcome and not the average marginal effect of a variable across conditions—opens up the possibility to assess how quantitative estimation strategies fare when applied to estimating this estimand.

A second point of difference is nicely summarized by Goertz and Mahoney (2012, 230): “qualitative analysts adopt a ‘causes-of-effects’ approach to explanation [... whereas] statistical researchers follow the ‘effects-of- causes’ approach employed in experimental research.” We agree with this association, though from a MIDA perspective we see such distinctions as differences in estimands and not as differences in ontology. Conditioning on a given *X* and *Y* the effects-of-cause question is *E(Yi(Xi =* 1) − *Y*_*i*_(*X*_*i*_ *=* 0)). By contrast, the cause-of- effects question can be written Pr(*Y*_*i*_(0) = 0| *X*_*i*_ = 1, *Y*_*i*_(1) = 1). This expression asks what are the chances that *Y* would have been 0 if *X* were 0for aunit *i* for which *X* was 1 and *Y*_i_(1) was 1. The two questions are of a similar form though the cause-of-effects question is harder to answer (Dawid 2000). Once thought of as questions about what the estimand is, one can assess directly when one or another estimation strategy is more or less effective at facilitating inference about the estimand of interest. In fact, experiments are in general not able to solve the identification problem for cause-of-effects questions (Dawid 2000) and this may be one reason for why these questions are often ignored by quantitative researchers. Exceptions include Yamamoto (2012) and Balke and Pearl (1994).

Below, we demonstrate gains from declaration of designs in a common framework by providing examples of design declaration for crisp-set qualitative comparative analysis (Ragin 1987), nested case analysis (Lieberman 2005), and CPO (causal process observation) process-tracing (Collier 2011; Fairfield 2013), alongside experimental and quasi-experimental designs.

Overall, this discussion suggests that the common ways in which designs are conceptualized produce three distinct problems. First, the different components of a design may not be chosen to work optimally together. Second, consideration is unevenly distributed across components of a design. Third, the absence of a common framework across research traditions obscures where the points of overlap and difference lie and may limit both critical assessment of approaches and cross-fertilization. We hope that the MIDA framework and tools can help address these challenges.

## DECLARING AND DIAGNOSING RESEARCH DESIGNS IN PRACTICE

A design that can be declared in computer code can then be simulated in order to diagnose its properties. The approach to declaration that we advocate is one that conceives of a design as a concatenation of steps. To illustrate, the top panel of Table 3 shows how to declare a design in code using the companion software to this paper, DeclareDesign (Blair etal. 2018). The resulting set of objects (p _U, f_Y, I, p _S, p _Z, R, and A) are all steps. Formally, each of these steps is a function. The design is the concatenation of these, which we represent using the “ + ” operator: design <– p _U + f _Y + I + p _S + p _Z + R + A. A single simulation runs through these steps, calling each of the functions successively. A design diagnosis conducts *m* simulations, then summarizes the resulting distribution of diagnostic statistics in order to estimate the diagnosand.

Diagnosands can be estimated with higher levels of precision by increasing *m*. However, simulations are often computationally expensive. In order to assess whether researchers have conducted enough simulations to be confident in their diagnosand estimates, we recommend estimating the sampling distributions of the diagnosands via the nonparametric bootstrap.^11^ With the estimated diagnosand and its standard error, we can characterize our uncertainty about whether the range of likely values of the diagnosand compare favorably to reference values such as statistical power of 0.8.^12^

Design diagnosis places a burden on researchers to come up with a causal model, M. Since researchers presumably want to learn about the model, declaring it in advance may seem to beg the question. Yet declaring a model is often unavoidable when diagnosing designs. In practice, doing so is already familiar to any researcher who has calculated the power of a design, which requires the specification of effect sizes. The seeming arbitrariness of the declared model can be mitigated by assessing the sensitivity of diagnosis to alternative models and strategies, which is relatively straightforward given a diagnosand-complete design declaration. Further, researchers can inform their substantive models with existing data, such as baseline surveys. Just as power calculators focus attention on minimum detectable effects, design declaration offers a tool to demonstrate design properties and how they change depending on researcher assumptions.

In the next sections, we illustrate how research designs that aim to answer descriptive, causal, and exploratory research questions can be declared and diagnosed in practice. We then describe how the estimand-focused approach we propose works with designs that focus less on estimand estimation and more on modeling data generating processes. In all cases, we highlight potential gains from declaring designs using the MIDA framework.

### Descriptive Inference

Descriptive research questions often center on measuring a parameter in a sample or in the population, such as the proportion of voters in the United States who support the Democratic candidate for president. Although seemingly very different from designs that focus on causal inference, because of the lack of explanatory variables, the formal differences are not great.

#### Survey Designs

We examine an estimator of candidate support that conditions on being a “likely voter.” For this problem, the data that help researchers predict who will vote are of critical importance. In the Supplementary Materials Section 3.1, we declare a model in which latent voters are likely to vote for a candidate, but overstate their true propensity to vote. The inquiry is the true underlying support for the candidate among those who will vote, while the data strategy involves taking a random sample from the national adult population and asking survey questions that measure vote intention and likelihood of voting. As an answer strategy, we estimate support for the candidate among likely voters. The diagnosis shows that when people misreport whether they vote, estimates of candidate support may be biased, a commonplace observation about the weaknesses of survey measures. The utility of design declaration here is that we can calibrate how far off our estimates will be under reasonable models of misreporting.

#### Bayesian Descriptive Inference

Although our simulation approach has a frequentist flavor, the MIDA framework itself can also be applied to Bayesian strategies. In Supplementary Materials Section 3.2, we declare a Bayesian descriptive inference design. The model stipulates a latent probability of success for each unit, and makes one binomial draw for each according to this probability. The inquiry is the true latent probability, and the data strategy involves a random sample of relatively few units. We consider two answer strategies: first, we stipulate uniform priors, with a mean of 0.50 and a standard deviation of 0.29; in the second, we place more prior probability mass at0.50, with a standard deviation of 0.11.

Once declared, the design can be diagnosed not only in terms of its bias, but also as a function of quantities specific to Bayesian estimation approaches, such as the expected shift in the location and scale of the posterior distribution relative to the prior distribution. The diagnosis shows that the informative prior approach yields more certain and more biased inferences than the uniform prior approach. In terms of the bias-variance tradeoff, the informative priors decrease the posterior standard deviation by 40% relative to the uniform priors, but increase the bias by 33%.

### Causal Inference

The approach to design diagnosis we propose can be used to declare and diagnose a range of research designs typically employed to answer causal questions in the social sciences.

#### Process Tracing

Although not all approaches to process tracing are readily amenable to design declaration (e.g., theory-building process tracing, see Beach and Pedersen 2013, 16), some are. We focus here on Bayesian frameworks that have been used to describe process tracing logics (e.g., Bennett 2015; Humphreys and Jacobs 2015; Fairfield and Charman 2017). In these approaches, “causal process observations” (CPOs) are believed to be observed with different probabilities depending on the causal process that has played out in a case. Ideal-type CPOs as described by Van Evera (1997)are “hoop tests” (CPOs that are nearly certain to be seen if the hypothesis is true, but likely either way), “smoking-gun tests” (CPOs that are unlikely to be seen in general but are extremely unlikely if a hypothesis is false), and “doubly- decisive tests” (CPOs that are likely tobe seen if and only if a hypothesis is true)^13^. Unlike much quantitative inference, such studies often pose “causes-of-effects” inquiries (did the presence of a strong middle class cause a revolution?), and not “effects-of-causes” questions (what is the average effect of a strong middle class on the probability of a revolution happening?) (Goertz and Mahoney 2012). Such inquiries often imply a hypothesis— “the strong middle class caused the revolution,” say—that can be investigated using Bayes’ rule.

Formalizing this kind of process-tracing exercise leads to non-obvious insights about the tradeoffs involved in committing to one or another CPO strategy ex ante. We declare a design based on a model of the world in which both the driver, *X*, and the outcome, *Y*, might be present in a given case either because *X* caused *Y* or because *Y* would have been present regardless of *X* (or perhaps, an alternative cause was responsible for Y). See Supplementary Materials Section 3.3. The inquiry is whether *X* in fact caused *Y* in the specific case under analysis (i.e., would *Y* have been different if *X* were different?). The data strategy consists of selecting one case from a population of cases, based on the fact that both *X* and *Y* are present, and then collecting two causal process observations. Even before diagnosis, the declaration of the design illustrates an important point: the case selection strategy informs the answer strategy by enabling the researcher to narrow down the number of causal processes that might be at play. This greatly simplifies the application of Bayes’ rule to the case in question.

Importantly, the researcher attaches two different ex ante probabilities to the observation of confirmatory evidence in each CPO, depending on whether *X* did or did not cause *Y*. Specifically, the first CPO contains evidence that is more likely to be seen when the hypothesis is true, Pr(E_1_|H) = 0.75, but even when *H* is false and *Y* happened irrespective of *X*, there is some probability of observing the first piece of evidence: Pr(E_1_|¬H) = 0.25. The first CpO thus constitutes a “straw-in-the-wind” test (albeit a reasonably strong one). By contrast, the probability of observing the evidence in the second CPO when the hypothesis that *X* caused *Y* is true, Pr(E_2_|H) is 0.30, whereas the probability of observing the evidence when the hypothesis is false, Pr(E_2_|¬H) is only 0.05. The second CPO thus constitutes a “smoking gun” test of *H*. Observing the second piece of evidence is more informative than observing the first, because it is so unlikely to observe a smoking gun when the hypothesis is false.

Diagnosis reveals that a researcher who relied solely on the weaker “straw-in-the-wind” test would make *better* inferences on average than one who relied solely on the “smoking gun” test. One does better relying on the straw because, even if it is less informative when observed, it is much more commonly observed than the smoking gun, which is an informative, but rare, clue. The Collier (2011, 826) assertion that, of the four tests, straws-in-the-wind are “the weakest and place the least demand on the researcher’s knowledge and assumptions” might thus be seen as an advantage rather than a disadvantage. In practice, of course, scholars often seek multiple CPOs, possibly of different strength (see, for example, Fairfield 2013). In such cases, the diagnosis suggests the learning depends on the ways in which these CPOs are correlated. There are large gains from seeking two CPOs when they are negatively correlated— for example, if they arise from alternative causal processes. But there are weak gains when CPOs arise from the same process. Presentations of process tracing rarely describe correlations between CPO probabilities yet the need to specify these (and the gain from doing so) presents itself immediately when a process tracing design is declared.

#### Qualitative Comparative Analysis (QCA)

One approach to mixed methods research focuses on identifying ways that causes combine to produce outcomes. What, for instance, are the combinations of demography, natural resource abundance, and institutional development that give rise to civil wars? An answer might be of the form: conflicts arise when there is natural resource abundance *and* weak institutional structure *or* when there are deep ethnic divisions. The key idea is that different configurations of conditions can lead to the same outcome (equifinality) and the interest is in assessing which combinations of conditions matter.

Many applications of qualitative comparative analysis use Boolean minimization algorithms to assess which configurations of factors are associated with different outcomes. Critics have highlighted that these algorithms are sensitive to measurement error (Hug 2013). Pointing to such sensitivity, some even go as far as to call for the rejection of QCA as a framework for inquiry (Lucas and Szatrowski 2014; for a nuanced response, see Baumgartner and Thiem 2017).

However, a formal declaration of a QCA design makes clear that these criticisms unnecessarily conflate QCA answer strategies with their inquiries (for a similar argument, see Collier 2014). Contrary to claims that regression analysis and QCA stem from fundamentally different ontologies (Thiem, Baumgartner, and Bol 2016), we show that saturated regression analysis may mitigate measurement error concerns in QCA. This simple proof of concept joins efforts toward unifying QCA with aspects of mainstream statistics (Braumoeller 2003; Rohlfing 2018) and other qualitative approaches (Rohlfing and Schneider 2018).

In Supplementary Materials Section 3.4 we declare a QCA design, focusing on the canonical case of binary variables (“crisp-set QCA”). The model features an outcome *Y* that arises in a case if and only if cause *A* is absent *and* cause *B* is present *(Y = a* * B). The approach extends readily to cases with many causes in complex configurations. For our inquiry, we wish to know the true minimal set of configurations of conditions that are sufficient to cause *Y*. The data strategy involves measuring and encoding knowledge about *Y*in a truth table. We allow for some error in this process. As in Rohlfing (2018), we are agnostic as to how this error arises: it may be that scholarly debate generates epistemic uncertainty about whether *Y* is truly present or absent in a given case, or that there is measurement error due to sampling variability.

For answer strategies, we compare two QCA minimization approaches. The first employs the classical Quine-McCluskey (QMC) minimization algorithm (see Duşa and Thiem 2015, for a definition) and the second the “Consistency Cubes” (CCubes) algorithm (Duşa 2018) to solve for the set of causal conditions that produces *Y*. This comparison demonstrates the utility of declaration and diagnosis for researchers using QCA algorithms, who might worry about whether their choice of algorithm will alter their inferences.^14^ We show that, at least in simple cases such as this, such concerns are minimal.

We also consider how ordinary least squares minimization performs when targeting a QCA estimand. The right hand side of the regression includes indicators for membership in all feasible configurations of ***A*** and B. Configurations that predict the presence of *Y* with probability greater than 0.5 are then included in the set of sufficient conditions.

The diagnosis of this design shows that QCA algorithms can be successful at pinpointing exactly the combination of conditions that give rise to outcomes. When there is no error and the sample is large enough to ensure sufficient variation in the data, QMC and CCubes successfully recover the correct configuration 100% of the time. The diagnosis also confirms that QCA via saturated regression can recover the data generating process correctly and the configuration of causes esti- mand can then be computed, correctly, from estimated marginal effects.

This last point is important for thinking through the gains from employing the MIDA framework. The declaration clarifies that QCA is not equivalent to saturated regression: without substantial transformation, regression does not target the QCA estimands (Thiem, Baumgartner, and Bol 2016). However, it also clarifies that regression models *can* be integrated into classical QCA inquiries, and do very well. Using regression to perform QCA is equivalent to QMC and CCubes when there is no error, and even slightly outperforms these algorithms (on the diagnosands we consider) in the presence of measurement error. More work is required to understand the conditions under which the approaches perform differently.

However, the declaration and diagnosis illustrate that there need not be a tension between regression as an estimation procedure and causal configurations as an estimand. Rather than seeing them as rival research paradigms, scholars interested in QCA estimands can combine the machinery developed in the QCA literature to characterize configurations of conditions with the machinery developed in the broader statistical literature to uncover data generating processes. Thus, for instance, in answer to critiques that the method does not have a strategy for causal identification (Tanner 2014), one could in principle try to declare designs in which instrumental variables strategies, say, are used in combination with QCA estimands.

#### Nested Mixed Methods

A second approach to mixed methods research nests qualitative small *N* analysis within a strategy that involves movement back and forwards between large *N* theory testing and small *N* theory validation and theory generation. Lieberman (2005) describes a strategy of nested analysis of this form. In Supplementary Materials Section 3.5, we specify the estimands and analysis strategies implied by the procedure proposed in Lieberman (2005). In our declaration, we assume a model with binary variables and an inquiry focused on the relationship between *X* and *Y* (both causes-of-effects and effects-of-causes are studied). The model allows for the possibility that there are variables that are not known to the researcher when conducting large *N* analysis, but might modify or confound the relationship between *X* and *Y*. The data strategy and answer strategies are quite complex and integrated with each other. The researcher begins by analyzing a data set involving *X* and *Y*. If the quantitative analysis is “successful” (defined in terms of sufficient residual variance explained), the researcher engages in within- case “on the regression line” analysis. Using within-case data, the researcher assesses the extent to which *X* plausibly caused *Y* (or not *X* caused not *Y*) in these cases. If the qualitative or quantitative analyses reject the model, then a new qualitative analysis is undertaken to better understand the relationship between *X* and *Y*. In the design, this qualitative exploration is treated as the possibility of discovering the importance of a third variable that may moderate the effect of *X* on *Y*. If an alternative model is successfully developed, it is then tested on the same large *N* data.

Diagnosis of this design illustrates some of its advantages. In particular, in some settings the within- case analysis can guide researchers to models that better capture data generating processes and improve identification. The declaration also highlights the design features that are left to researchers. How many cases should be gathered and how should they be selected? What thresholds should be used to decide whether a theory is successful or not? The design diagnosis suggests interesting interactions between these design elements. For instance, if the bar for success in the theory testing stage is low in terms of the minimum share of cases explained that are considered adequate, then the researcher might be better off sampling fewer qualitative cases in the testing stage and more in the development stage. More variability in the first stage makes it more likely that one would reject a theory, which might in turn lead to the discovery of a better theory.

#### Observational Regression-Based Strategies

Many observational studies seek to make causal claims, but do not explicitly employ the potential outcomes framework, instead describing inquiries in terms of model parameters. Sometimes studies describe their goal as the estimation of a parameter b from a model of the form *y*_*i*_ *= α* + *βx*_*i*_ + ε_*i*_. What is the estimand here? If we believe that this model describes the true data generating process, then *β is* an estimand: it is the true (constant) marginal effect of *x* on *y*. But what if we are wrong about the model? We run into a problem if we want to assess the properties of strategies under different assumptions about data generation when the inquiry itself depends on the data generating model.

To address this problem, we can declare an inquiry as a summary of differences in potential outcomes across conditions, *β*. Such a summary might derive from a simple comparison of potential outcomes—for example $\tau \equiv E_{x}E_{i}\left(Y_{i}(x)-Y_{i}(x-1)\right)$ captures the difference in outcomes between having income *x* and having a dollar less, *x* − 1, for different possible income levels. Or it could be a parameter from a model applied to the potential outcomes. For example we might define *α* and *β* as the solutions to:

$$\underset{\left(\alpha ,\beta \right)}{\min}\;\sum_{i}\int {\left(Y_{i}(x)-\alpha -\beta x\right)}^{2}f(x)\text{d}x$$

Here *Y*_*i*_(*x*) is the (unknown) potential outcome for unit *i* in condition *x*. Estimand *β* can be thought of as the coefficient one would get on *x* if one were to able to regress all possible potential outcomes on all possible conditions for all units (given density of interest *f*(*x*)).^15^ Our data strategy will simply consist of the passive observation of units in the population, and we assess the performance of an answer strategy employing an OLS model to estimate *β* under different conditions.

To illustrate, we declare a design that lets us quickly assess the properties of a regression estimate under the assumption that in the true data-generating process ***y*** is in fact a nonlinear function of *x* (Supplementary Materials Section 3.6). Diagnosis of the design shows that under uniform random assignment of *x*, the linear regression returns an unbiased estimate of a (linear) estimand, even though the true data generating process is nonlinear. Interestingly, with the design in hand, it is easy to see that unbiasedness is lost in a design in which different values of *x*_*i*_ are assigned with differing probabilities. The benefit of declaration here is that, without defining *I*, it is hard to see the conditions under which *A* is biased or unbiased. Declaration and diagnosis clarify that, even though the answer strategy “assumes” a nonlinear relationship in *M* that does not hold, under certain conditions OLS is still able to estimate a linear summary of that relationship.

#### Matching on Observables

In many observational research designs, the processes by which units are assigned to treatment are not known with certainty. In matching designs, the effects of unknown assignment procedure may, for example, be assessed by matching units on their observable traits under an assumption of as-if random assignment between matched pairs. Diagnosis in such instances can shed light on risks when such assumptions are not justified. In Supplementary Materials Section 3.7, we declare a design with a model in which three observable random variables are combined in a probit process that assigns the treatment variable, *Z*. The inquiry pertains to the average treatment effect of *Z* on the outcome *Y* among those actually assigned to treatment, which we estimate using an answer strategy that tries to reconstruct the assignment process to calculate *a*^*A*^. Our diagnosis shows that matching improves mean-squared- error (*E*[(*a*^*A*^ − *a*^*M*^)^2^]) relative to a naive difference-in-means estimator of the treatment effect on the treated (ATT), but can nevertheless remain biased (E[*a*^*A*^ − *a*^*M*^] ≠ 0) if the matching algorithm does not successfully pair units with equal probabilities of assignment, i.e., if matching has not eliminated all sources of confounding. The chief benefit of the MIDA declaration here is to separate out beliefs about the data generating process (*M*) from the details of the answer strategy (*A*), whose robustness to alternative data generating processes can then be assessed.

#### Regression Discontinuity

While in many observational settings researchers do not know the assignment process, in others, researchers may know how assignment works without necessarily controlling it. In regression discontinuity designs, causal identification is often premised on the claim that potential outcomes are continuous at a critical threshold (see De la Cuesta and Imai 2016; Sekhon and Titiunik 2016). The declaration of such designs involves a model that defines the unknown potential outcomes functions mapping average outcomes to the running and treatment variables. Our inquiry is the difference in the conditional expectations of the two potential outcomes functions at the discontinuity. The data strategy involves passive observation and collection of the data. The answer strategy is a polynomial regression in which the assignment variable is linearly interacted with a fourth order polynomial transformation of the running variable. In Supplementary Materials Section 3.8, we declare and diagnose such a design.

The declaration highlights a difference between this design and many others: the estimand here is not an average of potential outcomes of a set of sample units, but rather an unobservable quantity defined at the limit of the discontinuity. This feature makes the definition of diagnosands such as bias or external validity conceptually difficult. If researchers postulate unobservable counter-factuals, such as the “treated” outcome for a unit located below the treatment threshold, then the usefulness of the regression discontinuity estimate of the average treatment effect for a specific set of units can be assessed.

#### Experimental Design

In experimental research, researchers are in control of sample construction and assignment of treatments, which makes declaring these parts of the design straightforward. A common choice faced in experimental research is between employing a 2-by-2 factorial design or a three-arm trial where the “both” condition is excluded. Suppose we are interested in the effect of each of two treatments *when the other condition is set to control.* Should we choose a factorial design or a three- arm design? Focusing for simplicity on the effect of a single treatment, we declare two designs under a range of alternative models to help assess the tradeoffs. For both designs, we consider models *M*_*1*_,..., *M*_*K*_, where we let the interaction between treatments vary over the range −0.2 to +0.2. Our inquiry is always the average treatment effect of treatment 1 given all units are in the control condition for treatment 2. We consider two alternative data strategies: an assignment strategy in which subjects are assigned to a control condition, treatment 1, or treatment 2, each with probability 1/3; and an alternative strategy in which we assign subjects to each of four possible combinations of factors with probability 1/4. The answer strategy in both cases involves a regression of the outcome on both treatment indicators with no interaction term included.

**FIGURE 1.**
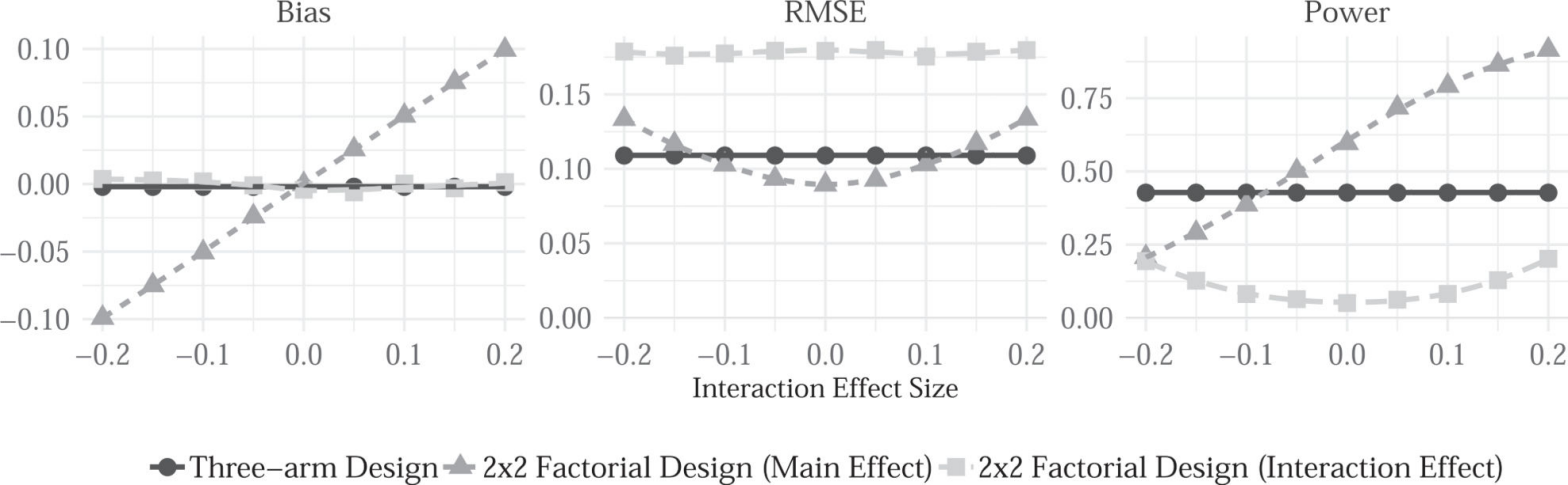
Diagnoses of Designs With Factorial or Three-Arm Assignment Strategies Illustrate a Bias-Variance Tradeoff Bias (left), root mean-squared-error (center), and power (right) are displayed for two assignment strategies, a 2 × 2 treatment arm factorial design (black solid lines; circles) and a three-arm design (gray dashed lines; triangles) according to varying interaction effect sizes specified in the potential outcomes function (*x* axis). The third panel also shows power for the interaction effect (squares) from the factorial design.

We declare and diagnose this design and confirm that neither design exhibits bias when the true interaction term is equal to zero (Figure 1 left panel). The details of the declaration can be found in Supplementary Materials Section 3.9. However, when the interaction between the two treatments is stronger, the factorial design renders estimates of the effect of treatment 1 that are more and more biased relative to the “pure” main effect estimand. Moreover, there is a bias-variance tradeoff in choosing between the two designs when the interaction is weak (Figure 1 right panel). When the interaction term is close to zero, the factorial design is preferred, because it is more powerful: it compares one half of the subject pool to the other half, whereas the three-arm design only compares a third to a third. However, as the magnitude of the interaction term increases, the precision gains are offset by the increase in bias documented in the left-panel. When the true interaction between treatments is large, the three-arm design is then preferred. This exercise highlights key points of design guidance. Researchers often select factorial designs because they expect interaction effects, and indeed factorial designs are required to assess these. However if the scientific question of interest is the pure effect of each treatment, researchers should (perhaps counterintuitively) use a factorial design if they expect *weak* interaction effects. An integrated approach to design declaration here illustrates non-trivial interactions between the ***d***ata strategy, on the one hand, and the ability of answers *(a*^*A*^*)* to approximate the estimand (*a*^*M*^), on the other.

### Designs for Discovery-Oriented Research

In some research projects, the ultimate hypotheses that are assessed are not known at the design stage. Some inductive designs are entirely unstructured and explore a variety of data sources with a variety of methods within a general domain of interest until a new insight is uncovered. Yet many can be described in a more structured way.

In studying textual data, for example, a researcher may have a procedure for discovering the “topics” that are discussed in a corpus of documents. Before beginning the research, the set of topics and even the number of topics is unknown. Instead, the researcher selects a model for estimating the content of a fixed number of topics (e.g., Blei, Ng, and Jordan 2003) and a procedure for evaluating the model fit used to select which number of topics fits the data best. Such a design is inductive, yet the analytical discovery process can be described and evaluated.

We examine a data analysis *procedure* in which the researcher assesses possible analysis strategies in a first stage on half of the data and in the second stage applies her preferred procedure to the second half of the data. Split-sample procedures such as this enable researchers to learn about the data inductively while protecting against Type I errors (for an early discussion of the design, see Cox 1975). In Supplementary Materials Section 3.10, we declare a design in which the model stipulates a treatment of interest, but also specifies groups for which there might be heterogeneous treatment effects. The main inquiry pertains to the treatment effect, but the researchers anticipate that they may be interested in testing for heterogeneous treatment effects if they observe prima facie evidence for it. The data strategy involves random assignment. The answer strategy involves examination of main effects, but in addition the researchers examine heterogeneous treatment effects inside a random subgroup of the data. If they find evidence of differential effects they specify a new inquiry which is assessed on the remaining data. The results on heterogeneous effects are compared against a strategy that simply reports discoveries found using complete data, rather than on split data (we call this the “unprincipled” approach).

We see lower bias from principled discovery than from unprincipled discovery as one might expect. The declaration and diagnosis also highlight tradeoffs in terms of mean squared error. Mean squared error is not necessarily lower for the principled approach since fewer data are used in the final test. Moreover, the principled strategy is somewhat less likely to produce a result *at all* since it is less likely that a result would be discovered in a subset of the data than in the entire data set. With this design declared, one can assess what an optimal division of units into training and testing data might be given different hypothesized effect sizes.

### Designs for Modeling Data Generation Processes

For most designs we have described, the estimand of interest is a number: an average level, a causal effect, or a summary of causal effects. Yet in some situations, researchers seek not to estimate a particular number, but rather to model a data generating process. For work of this kind, the data generating process ***is*** the estimand, rather than any particular comparison of potential outcomes. This was the case for the qualitative QCA design we looked at, in which the combination of conditions that produce an outcome was the estimand. This model- focused orientation is also common for quantitative researchers. In the example from Observational Regression-Based Strategies, we noted that a researcher might be interested not in the average effect resulting from a change in *X* over some range, but in estimating a function $f_{Y}^{*}(X)$ (which itself might be used to learn about different quantities of interest). This kind of approach can be handled within the MIDA framework in two ways. One asks the researcher to identify the ultimate quantities of interest *ex ante* and to treat these as the estimands. In this case, the model generated to make inferences about quantities of interest is thought of as part of the answer strategy, *a,* and not part of *i*. A second approach posits a true underlying DGP as part of *m*, $f_{Y}^{**}$. The estimand is then also a function, $f_{Y}^{*}$, which could be $f_{Y}^{**}$ itself or an approximation.^16^ An estimate is a function *f*_*Y*_ that aims to approximate $f_{Y}^{*}$. In this case, it is difficult to think of diagnosands like bias or coverage when comparing $f_{Y}^{*}$ to *f*_*Y*_, but diagnosands can still be constructed that measure the success of the modeling. For instance, for a range of values of *X* we could compare values of *f*_*Y*_(*X*) to $f_{Y}^{*}(X)$, or employ familiar statistics of goodness of fit, such as the *R*^2^. The MIDA framework forces clarity regarding which of these approaches a design is using, and as a consequence, what kinds of criticisms of a design are on target. For instance, returning to the regression strategies example: if a linear model is used to estimate a linear estimand, it may behave well for that purpose even when the underlying process is very nonlinear. If, however, the goal is to estimate the shape of the data generating process, the linear estimator will surely fare poorly.

The research designs we have described in this section are varied in the intellectual traditions as well as inferential goals they represent. Yet commonalities emerge, which enabled us to declare each design in terms of MIDA. Exploring this broad set of research practices through MIDA clarified non-obvious aspects of the designs, such as the target of inference (Inquiry) in QCA designs or regression discontinuity designs with finite units, as well as the subtle implications of beliefs about heterogeneity in treatment effects (Model) for selecting between three-arm and 2 × 2 factorial designs.

## PUTTING DECLARATIONS AND DESIGN DIAGNOSIS TO USE

We have described and illustrated a strategy for declaring research designs for which “diagnosands” can be estimated given conjectures about the world. How might declaring and diagnosing research designs in this way affect the practices of authors, readers, and replication authors? We describe implications for how designs are chosen, communicated, and challenged.

### Making Design Choices

The move toward increasing credibility of research in the social sciences places a premium on considering alternative data strategies and analysis strategies at early stages of research projects, not only because it reduces researcher discretion after observing outcomes, but more importantly because it can improve the quality of the final research design. While there is nothing new about the idea of determining features such as sampling and estimation strategies ex ante, in practice many designs are finalized late in the research process, after data are collected. Frontloading design decisions is difficult not only because existing tools are rudimentary and often misleading, but because it is not clear in current practice what features of a design must be considered ex ante.

We provide a framework for identifying *which* features affect the assessment of a design’s properties, declaring designs and diagnosing their inferential quality, and frontloading design decisions. Declaring the design’s features in code enables direct exploration of alternative data and analysis strategies using simulated data; evaluating alternative strategies through diagnosis; and exploring the robustness of a chosen strategy to alternative models. Researchers can undertake each step before study implementation or data collection.

### Communicating Design Choices

Bias in published results can arise for many reasons. For example, researchers may deliberately or in advertently select analysis strategies because they produce statistically significant results. Proposed solutions to reduce this kind of bias focus on various types of preregistration of analysis strategies by researchers (Casey, Glennerster, and Miguel 2012; Green and Lin 2016; Nosek et al. 2015; Rennie 2004; Zarin and Tse 2008). Study registries are now operating in numerous areas of social science, including those hosted by the American Economic Association, Evidence in Governance and Politics, and the Center for Open Science. Bias may also arise from reviewers basing publication recommendations on statistical significance. Results- blind review processes are being introduced in some journals to address this form of bias (e.g., Findley et al. 2016).

However, the effectiveness of design registries and results-blind review in reducing the scope for either form of publication bias depends on clarity over which elements must be included to describe the design. In practice, some registries rely on checklists and preanalysis plans exhibit great variation, ranging from lists of written hypotheses to all-but-results journal articles. In our view, the solution to this problem does not lie in ever-more-specific questionnaires, but rather in a new way of characterizing designs whose analytic features can be diagnosed through simulation.

The actions to be taken by researchers are described by the data strategy and the answer strategy; these two features of a design are clearly relevant elements of a preregistration document. In order to know which design choices were made ex ante and which were arrived at ex post, researchers need to communicate their data and answer strategies unambiguously. However, assessing whether the data and answer strategies are any good usually requires specifying a model and an inquiry. Design declaration can clarify for researchers and third parties what aspects of a study need to be specified in order to meet standards for effective preregistration. Rather than asking: “are the boxes checked?” the question becomes: “can it be diagnosed?” The relevant diagnosands will likely depend on the type of research design. However, if an experimental design is, for example, “bias complete,” then we know that sufficient information has been given to define the question, data, and answer strategy unambiguously.

Declaration of a design in code also enables a final and infrequently practiced step of the registration process, in which the researcher “reports and reconciles” the final with the planned analysis. Identifying how and whether the features of a design diverge between ex ante and ex post declarations highlights deviations from the preanalysis plan. The magnitude of such deviations determines whether results should be considered exploratory or confirmatory. At present, this exercise requires a review of dozens of pages of text, such that differences (or similarities) are not immediately clear even to close readers. Reconciliation of designs declared in code can be conducted automatically, by comparing changes to the code itself (e.g., a move from the use of a stratified sampling function to simple random sampling) and by comparing key variables in the design such as sample sizes.

### Challenging Design Choices

The independent replication of the results of studies after their publication is an essential component of the shift toward more credible science. Replication — whether verification, reanalysis of the original data, or reproduction using fresh studies — provides incentives for researchers to be clear and transparent in their analysis strategies, and can build confidence in findings.^17^

In addition to rendering the design more transparent, diagnosand-complete declaration can allow for a different approach to the re-analysis and critique of published research. A standard practice for replicators engaging in reanalysis is to propose a range of alternative strategies and assess the robustness of the *data- dependent* estimates to different analyses. The problem with this approach is that, when divergent results are found, third parties do not have clear grounds to decide which results to believe. This issue is compounded by the fact that, in changing the analysis strategy, replicators risk departing from the estimand of the original study, possibly providing different answers to different questions. In the worst case scenario, it can be difficult to determine what is learned both from the original study and from the replication.

A more coherent strategy facilitated by design simulations would be to use a diagnosand-complete declaration to conduct “design replication.” In a design replication, a scholar restates the essential design characteristics to learn about what the study *could have* revealed, not just what the original author reports *was* revealed. This helps to answer the question: under what conditions are the results of a study to be believed? By emphasizing abstract properties of the design, design replication provides grounds to support alternative analyses on the basis of the original authors’ intentions and not on the basis of the degree of divergence of results. Conversely, it provides authors with grounds to question claims made by their critics.

Table 4 illustrates situations that may arise. In a declared design an author might specify situation 1: a set of claims on the structure of the variables and their potential outcomes (the model) and an estimator (the answer strategy). A critic might then question the claims on potential outcomes (for example, questioning a nospillovers assumption) or question estimation strategies (for example, arguing for inclusion or exclusion of a control variable from an analysis), or both.

In this context, there are several possible criteria for admitting alternative answer strategies:

- **Home Ground Dominance.** If ex ante the diagnostics for situation 3 are better than for 1 then this gives grounds to switch to 3. That is, if a critic can demonstrate that an alternative estimation strategy outperforms an original estimation strategy even under the data generating process assumed by an original researcher, then they have strong grounds to propose a change in strategies. Conversely, if an alternative estimation strategy produces different results, conditional on the data, but does not outperform the original strategy given the original assumptions, this gives grounds to question the reanalysis.
- **Robustness to Alternative Models.** If the diagnostics in situation 3 are as good as in 1 but are better in situation 4 than in situation 2 this provides a robustness argument for altering estimation strategies. For example, in a design with heterogeneous probabilities by block, an inverse propensity-weighted estimator will do about as well as a fixed effects estimator in terms of bias when treatment effects are constant, but will perform better on this dimension when effects are heterogeneous.
- **Model Plausibility.** If the diagnostics in situation 1 are better than in situation 3, but the diagnostics in situation 4 are better than in situation 2, then things are less clear and the justification of a change in estimators depends on the plausibility of the different assumptions about potential outcomes.

The normative value or relative ranking of these criteria should be left to individual research communities. Without a declared design, in particular the model and inquiry, none of these criteria can be evaluated, complicating the defense of claims for both the critic and the original author.

## APPLICATION: DESIGN REPLICATION OF Björkman and Svensson (2009)

We illustrate the insights that a formalized approach to design declaration can reveal through an application to the design of Björkman and Svensson (2009), which investigated whether community-based monitoring can improve health outcomes in rural Uganda.

We conduct a “design replication:” using available information, we posit a Model, Inquiry, Data, and Answer strategy to assess properties of Björkman and Svensson (2009). This design replication can be contrasted with the kind of reanalysis of the study’s data that has been conducted by Donato and Garcia Mosqueira (2016) or the reproduction by Raffler, Posner, and Parkerson (2019) in which the experiment was conducted again.

The exercise serves three purposes: first, it sheds light on the sorts of insights the design can produce without using the original study’s data or code; second, it highlights how difficulties can arise from designs in which the inquiry is not well-defined; third, we can assess the properties of replication strategies, notably those pursued by Donato and Garcia Mosqueira (2016) and Raffler, Posner, and Parkerson (2019), in order to make clearer the contributions of such efforts.

In the original study, Björkman and Svensson (2009) estimate the effects of treatment on two important indicators: child mortality, defined as the number of deaths per 1,000 live births among under-5 year-olds (taken at the catchment-area-level) and weight-for-age *z*-scores, which are calculated by subtracting from an infant’s weight the median for their age from a reference population, and dividing by the standard deviation of that population. In the original design, the authors estimate a positive effect of the intervention on weight among surviving infants. They also find that the treatment greatly decreases child mortality.

We briefly outline the steps of our design replication here, and present more detail in Supplementary Materials Section 4.

We began by positing a model of the world in which unobserved variables, “family health” and “community health,” determine both whether infants survive early childhood and whether they are malnourished.

Our attempt to define the study’s inquiry met with a difficulty: the weight of infants in control areas whose lives would have been saved if they had been in the treatment is undefined (for a discussion of the general problem known as “truncation-by-death,” see Zhang and Rubin 2003). Unless we are willing to make conjectures about undefined states of the world (such as the control weight of a child who would not have survived if assigned to the control), we can only define the average difference in individuals’ potential outcomes for those children whose survival is unaffected by the treatment: *E*[Weight(*Z* = 1) − Weight(*Z* = 0)|Alive(*Z* = 0) = Alive(*Z* = 1) = 1].^18^

As in the original article we stratify sampling on catchment area and cluster-assign households in 25 of the 50 catchment areas to the intervention.

We estimate mortality at the cluster level and weight- for-age among living children at the household level, as in Björkman and Svensson (2009).

**FIGURE 2.**
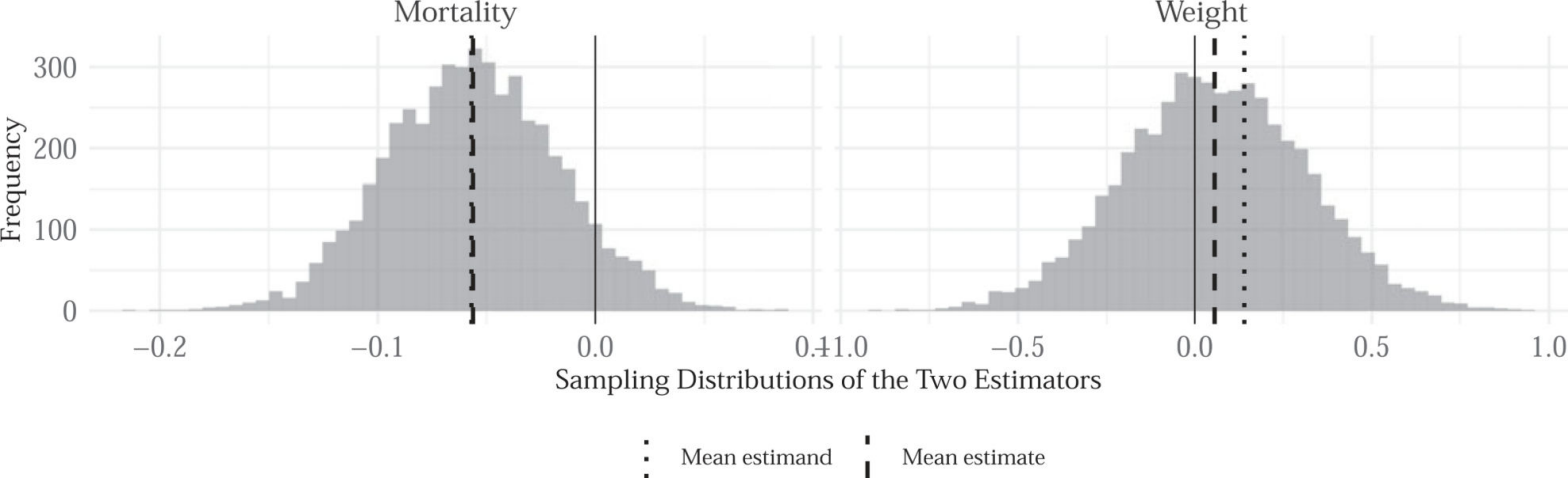
Data-independent Replication of Estimates in Björkman and Svensson (2009) Histograms display the frequency of simulated estimates of the effect of community monitoring on infant mortality (left) and on weight-for-age (right). The dashed vertical line shows the average estimate, the dotted vertical line shows the average estimand.

Figure 2 illustrates how the existence of an effect on mortality can pose problems for the unbiased estimation of an effect on weight-for-age. The histograms represent the sampling distributions of the average effect estimates of community monitoring on infant mortality and weight-for-age. The dotted vertical line represents the true average effect (*a*^*M*^). The mortality estimand is defined at the cluster level and the weight- for-age estimand is defined for infants who would survive regardless of treatment status. The dashed line represents the average answer, i.e., the answer we expect the design to provide (*E*[*a*^*A*^]). The weight-for-age answer strategy simply compares the weights of surviving infants across treatment and control. Under our postulated model of the world, the estimates of the effect on weight-for-age are biased downwards because it is precisely those infants with low health outcomes whose lives are saved by the treatment.

We draw upon the “robustness to alternative models” criterion (described in the previous section) to argue for an alternative answer strategy that exhibits less bias under plausible conjectures about the world.

An alternative answer strategy is to attempt to subset the analysis of the weight effects to a group of infants whose survival does not depend on the treatment. This approach is equivalent to the “find always-responders” strategy for avoiding post-treatment bias in audit studies (Coppock 2019). In the original study, for example, the effects on survival are much larger among infants younger than two years old. If indeed the survival of infants above this age threshold is unaffected by the treatment, then it is possible to provide unbiased estimates of the weight-for age effect, if only among this group. In terms of bias, such an approach does at least as well if we assume that there is no correlation between weight and mortality, and better if such a correlation does exist. It thus satisfies the “robustness to alternative models” criterion.

A reasonable counter to this replication effort might be to say that the alternative answer strategy does not meet the criterion of “home ground dominance” with respect to RMSE. The increase in variance from subsetting to a smaller group may outweigh the bias reduction that it entails. In both cases, transparent arguments can be made by formally declaring and comparing the original and modified designs.

The design replication also highlights the relatively low power of the weight-for-age estimator. As Gelman and Carlin (2014) have shown, conditioning on statistical significance in such contexts can pose risks of exaggerating the true underlying effect size. Based on our assumptions, what can we say here, specifically, about the risk of exaggeration? How effectively does a design such as that used in the replication by Raffler, Posner, and Parkerson (2019) mitigate this risk? To answer this question, we modify the sampling strategy of our simulation of the original study to include 187 clusters instead of 50.^19^ We then define the diagnosand of interest as the “exaggeration ratio” (Gelman and Carlin 2014): the ratio of the absolute value of the estimate to the absolute value of the estimand, given that the estimated effect is significant at the *α* ***=*** 0.05 level. This diagnosand thus provides a measure of how much the design exaggerates effect sizes conditional on statistical significance.

The original design exhibits a high exaggeration ratio, according to the assumptions employed in the simulations: on average, statistically significant estimates tend to exaggerate the true effect of the intervention on mortality by a factor of two and on weight-for-age by a factor of four. In other words, even though the study estimates effects on mortality in an unbiased manner, limiting attention to statistically significant effects provides estimates that are twice as large in absolute value as the true effect size on average. By contrast, using the same sample size as that employed in Raffler, Posner, and Parkerson (2019) reduces the exaggeration ratio on the mortality estimand to where it should be, around one.

Finally, we can also address the analytic replication by Donato and Garcia Mosqueira (2016). The replicators (D&M) noted that the eighteen community-based organizations who carried out the original “power to the people” (P2P) intervention were active in 64% of the treatment communities and 48% of the control communities. Donato and Garcia Mosqueira (2016) posit that prior presence of these organizations may be correlated with health outcomes, and therefore include in their analytic replication of the mortality and weight- for-age regressions both an indicator for CBO presence and the interaction of the intervention with CBO presence. The inclusion of these terms into the regression reduces the magnitude of the coefficients on the intervention indicator and thereby increases the *p*-values above the *α* ***=*** 0.1 threshold in some cases. The original authors (B&S) criticized the replicators’ decision to include CBO presence as a regressor, on the grounds that in any such study it is possible to find some unrelated variable whose inclusion will increase standard error of the treatment effect estimate.

In short, the original replicators make a set of contrasting claims about the true model of the world: B&S claim that CBO presence is unrelated to the outcome of interest (Björkman Nyqvist and Svensson 2016), whereas D&M claim that CBO presence might indeed affect (or be otherwise correlated with) health outcomes. As we argued in the previous section, diagnosis of the properties of the answer strategy under these competing claims should determine which answer strategy is best justified.

Since we do not know whether the replicators would have conditioned on CBO presence and its interaction with the intervention if it had not been imbalanced, we modify the original design to include four different replicator strategies: the first ignores CBO presence as in the original study; the second includes CBO presence irrespective of imbalance; the third includes an indicator for CBO presence only if the CBO presence is significantly imbalanced among the 50 treatment and control clusters at the *α* ***=*** 0.05 level; and the last strategy includes terms for both CBO presence and an interaction of CBO presence with the treatment irrespective of imbalance. We consider how these strategies perform under a model in which CBO presence is unrelated to health outcomes, and another in which, as claimed by the replicators, CBO presence is highly correlated with health outcomes.

Including the interaction term is a strictly dominated strategy from the standpoint of reducing mean squared error: irrespective of whether CBO presence is correlated with health outcomes or imbalanced, the RMSE expected under this strategy is higher than under any other strategy. Thus, based on a criterion of “Home Ground Dominance” in favor of B&S, one would be justified in discounting the importance of the replicators’ observation that “including the interaction term leads to a further reduction in magnitude and significance” of the estimated treatment effect (Donato and Garcia Mosqueira 2016,19).

Supposing now that there is no correlation between CBO presence and health outcomes, inclusion of the CBO indicator does increase RMSE ever so slightly in those instances where there is imbalance, and the standard errors are ever so slightly larger. On average, however, the strategies of conditioning on CBO presence regardless of balance and conditioning on CBO presence only if imbalanced perform about as well as a strategy of ignoring CBO presence when there is no underlying correlation. However, when there is a correlation between health outcomes and CBO presence, strategies that include CBO presence improve RMSE considerably, especially when there is imbalance. Thus, D&M could make a “Robustness to Alternative Models” claim in defense of their inclusion of the CBO dummy: including CBO presence does not greatly diminish inferential quality on average, even if there is no correlation in CBO presence and outcomes; and if there is such a correlation, including CBO presence in the regression specification strictly improves inferences. In sum, a diagnostic approach to replication clarifies that one should resist updating beliefs about the study based on the use of interaction terms, but that the inclusion of the CBO indicator only harms inferences in a very small subset of cases. In general, including it does not worsen inferences and in many cases can improve them. This approach helps to clarify which points of disagreement are most critical for how the scientific community should interpret and learn from replication efforts.

## CONCLUSION

We began with two problems faced by empirical social science researchers: selecting high quality designs and communicating them to others. The preceding sections have demonstrated how the MIDA framework can address both challenges. Once designs are declared in MIDA terms, diagnosing their properties and improving them becomes straightforward. Because MIDA describes a grammar of research designs that applies across a very broad range of empirical research traditions, it enables efficient sharing of designs with others.

Designing high quality research is difficult and comes with many pitfalls, only a subset of which are ameliorated by the MIDA framework. Others we fail to address entirely and in some cases, we may even exacerbate them. We outline four concerns.

The first is the worry that evaluative weight could get placed on essentially meaningless diagnoses. Given that design declaration includes declarations of conjectures about the world it is possible to choose inputs so that a design passes any diagnostic test set for it. For instance, a simulation-based claim to unbiasedness that incorporates all features of a design is still only good with respect to the precise conditions of the simulation (in contrast, analytic results, when available, may extend over general classes of designs). Still worse, simulation parameters might be selected because of their properties. A power analysis, for instance, may be useless if implausible parameters are chosen to raise power artificially. While MIDA may encourage more honest declarations, there is nothing in the framework that enforces them. As ever, garbage-in, garbage-out.

Second, we see a risk that research may get evaluated on the basis of a narrow, but perhaps inappropriate set of diagnosands. Statistical power is often invoked as a key design feature —but there may be little value in knowing the power of a study that is biased away from its target of inference. The appropriateness of the diagnosand depends on the purposes of the study. As MIDA is silent on the question of a study’s purpose, it cannot guide researchers or critics to the appropriate set of diagnosands by which to evaluate a design. An advantage of the approach is that the choice of diagnosands gets highlighted and new diagnosands can be generated in response to substantive concerns.

Third, emphasis on the statistical properties of a design can obscure the substantive importance of a question being answered or other qualitative features of a design. A similar concern has been raised regarding the “identification revolution” where a focus on identification risks crowding out attention to the importance of questions being addressed (Huber 2013). Our framework can help researchers determine whether a particular design answers a question well (or at all), and it also nudges them to make sure that their questions are defined clearly and *independently of their answer strategies*. It cannot, however, help researchers choose good questions.

Finally, we see a risk that the variation in the suitability of design declaration to different research strategies may be taken as evidence of the relative superiority of different types of research strategies. While we believe that the range of strategies that can be declared and diagnosed is wider than what one might at first think possible, there is no reason to believe that all strong designs can be declared either ex ante or ex post. An advantage of our framework, we hope, is that it can help clarify when a strategy can or cannot be completely declared. When a design cannot be declared, non-declarability is all the framework provides, and in such cases we urge caution in drawing conclusions about design quality.

We conclude on a practical note. In the end, we are asking that scholars add a step to their workflow. We want scholars to formally declare and diagnose their research designs both in order to learn about them and to improve them. Much of the work of declaring and diagnosing designs is already part of how social scientists conduct research: grant proposals, IRB protocols, preanalysis plans, and dissertation prospectuses contain design information and justifications for why the design is appropriate for the question. The lack of a common language to describe designs and their properties, however, seriously hampers the utility of these practices for assessing and improving design quality. We hope that the inclusion of a declaration and diagnosis step to the research process can help address this basic difficulty.

## Supplementary Material

Supplemental Materials

## Figures and Tables

**TABLE 1.** Examples of Diagnosands and the Elements of the Model (M), Inquiry (I), Data Strategy (D), and Answer Strategy (A) Required in Order for a Design to be Diagnosand-Complete for Each Diagnosand

		Required:			
Diagnosand	Description	M	I	D	A
Power	Probability of rejecting null hypothesis of no effect	✓		✓	✓
Estimation bias	Expected difference between estimate and estimand	✓	✓	✓	✓
Sampling bias	Expected difference between population average treatment effect and sample average treatment effect (Imai, King, and Stuart 2008)	✓	✓	✓	
RMSE	Root mean-squared-error	✓	✓	✓	✓
Coverage	Probability the confidence interval contains the estimand	✓	✓	✓	✓
SD of estimates	Standard deviation of estimates	✓		✓	✓
SD of estimands	Standard deviation of estimands	✓	✓	✓	
Imbalance	Expected distance of covariates across treatment conditions	✓		✓	
Type S rate	Probability estimate has incorrect sign, if statistically significant (Gelman and Carlin 2014)	✓	✓	✓	✓
Exaggeration ratio	Expected ratio of absolute value of estimate to estimand, if statistically significant (Gelman and Carlin 2014)	✓	✓	✓	✓
Value for money	Probability that a decision based on estimated effect yields net benefits	✓	✓	✓	✓
Robustness	Joint probability of rejecting the null hypothesis across multiple tests	✓		✓	✓

**TABLE 2.** Existing Tools Cannot Declare Many Core Elements of Designs and, as a Result, Can Only Calculate Some Diagnosands

(a) Declare design elements			(b) Diagnosis capabilities	
Design feature			Diagnosand	
(M)	Effect and block size correlated	0/30	Power (DIM estimator)	28/30
(I)	Estimand	0/30	Power (BFE estimator)	13/30
(D)	Sampling procedure	0/30	Power (IPW-BFE estimator)	0/30
(D)	Assignment procedure	0/30	Bias (*any* estimator)	0/30
(D)	Block sizes vary	1/30	Coverage (*any* estimator)	0/30
(A)	Probability weighting	0/30	SD of estimates (*any* estimator)	0/30

*Note:* Panel (a) indicates the number of tools that allow declaration of a particular feature of the design as part of the diagnosis. In the first row, for example, 0/30indicates that no tool allows researchers to declare correlated effect and block sizes. Panel (b) indicates the number of tools that can perform a particular diagnosis. Results correspond to design tool census concluded in July 2017 and do not include tools published since then.

**TABLE 3.** A Procedure for Declaring and Diagnosing Research Designs Using the Companion Software DeclareDesign (Blair et al. 2018)

	Design declaration	Code

$M\{\begin{array}{l} \mathrm{Declare}\;\mathrm{background}\;\mathrm{variables}\\ \mathrm{Declare}\;\mathrm{functional}\;\mathrm{relations} \end{array}$		p_U <-declare_population (N = 200, u = rnorm(N))
		f_Y <- declare potential outcomes(Y ~Z + u)
I	Declare inquiry	I <- declare_estimand (ATE=mean(Y_Z_1 − Y_Z_0))
$D\{\begin{array}{l} \mathrm{Declare}\;\mathrm{sampling}\\ \mathrm{Declare}\;\mathrm{assignment}\\ \mathrm{Declare}\;\mathrm{outcome}\;\mathrm{revelation} \end{array}$		p_S <-declare_sampling (n = 100) p_Z <- declare_assignment (m = 50)
		R <- declare_reveal (Y, Z)
A	Declare answer strategy	A <- declare estimator (Y ~Z, estimand = “ATE”)
	Declare design, (*M, I, D, A*)	design <- p_U + f_Y + I + p_S + p_Z + R + A

	Design simulation (1 draw)	Code

1	Draw a population *u* using *P(U)*	u <- p_U()
2	Generate potential outcomes using *f_Y_*	D <- f_Y(u)
3	Calculate estimand *a^M^*	a_M <- I(D)
4	Draw data, *d*, given model assumptions and data strategies	d <- R(p_Z(p_S(D)))
5	Calculate answers, *a*^*A*^ using *A* and *d*	a_A <- A(d)
6	Calculate a diagnostic statistic *t* using *a*^*A*^ and *a*^*M*^	e <- a_A[“estimate”] — a_M[“estimand”]

Design diagnosis (*m* draws)		Code

Declare a diagnosand		bias <- declare_diagnosands(bias = mean(estimate − estimand))
Calculate a diagnosand		diagnose_design(design_diagnosands = bias, sims = m)

*Note:* The top panel includes each element of a design that can be declared along with code used to declare them. The middle panel describes steps to simulate that design. The bottom panel includes the procedure to diagnose the design.

**TABLE 4.** Diagnosis Results Given Alternative Assumptions About the Model and Alternative Answer Strategies

	Author’s assumed model	Alternative claims on model
Author’s proposed answer strategy	1	2
Alternative answer strategy	3	4

*Note*: Four scenarios encountered by researchers and reviewers of a study are considered depending on whetherthe model orthe answer strategy differ from the author’s original strategy and model.

## Footnotes

1 Though *M* is a causal model of the world, such a model can be used for both causal and non-causal questions of interest.

2 The distinction lies in whether the conditional probability is recorded through passive observation or active intervention to manipulate the probabilities of the conditioning distribution. For example, Pr(*X*_1_|*X*_2_ = 1) might indicate the conditional probability that it is raining, given that Jack has his umbrella, whereas Pr(*X*_1_| *do*(*X*_2_ = 1))would indicate the probability of rain, given Jack is made to carry an umbrella.

3 See “Pre Analysis Plan Template” (60 features); World Bank Development Impact Blog (nine features).

4 Some exceptions are provided on page 4. Herron and Quinn (2016), for example, conduct a formal investigation of the RMSE and bias exhibited by the alternative case selection strategies proposed in an influential piece by Seawright and Gerring (2008).

5 We assessed tools listed in four reviews of the literature (Greenand MacLeod 2016; Groemping 2016; Guo et al. 2013; Kreidler et al., 2013),in addition to the first thirty results from Google searches of the terms “statistical bias calculator,” “statistical power calculator,” and “sample size calculator.” We found no admissible tools using the term “statistical bias calculator.” Thirty of the 143 tools we identified were able to diagnose inferential properties of designs, such as their power. See Supplementary Materials Section 2 for further details on the tool survey.

6 For example, no design could account for: the posited correlation between block size and potential outcomes; the sampling strategy; the exact randomization procedure; the formal definition of the estimand as the population average treatment effect; or the use of inverse- probability weighting. The one tool (GLIMMPSE) that was able to account for the blocking strategy encountered an error and was unable to produce diagnostic statistics.

7 Aronow and Samii (2016) express a similar concern for models using regression with controls.

8 Schneider and Wagemann (2012,320–1) also note that there are not grounds to assume incommensurability, noting that “if set-theoretic, method-specific concepts.... can be translated into the potential outcomes framework, the communication between scholars from different research traditions will be facilitated.” See also Mahoney (2008) on the consistency of these conceptualizations.

9 An INUS condition is “an insufficient but non-redundant part of an unnecessary but sufficient condition” (Mackie 1974).

10 Goertz and Mahoney (2012, 59) also make the point that the difference is in practice, and is not fundamental: “Within quantitative research, it does not seem useful to group cases according to common causal configurations on the independent variables. *Although one could do this,* it is not a practice within the tradition.” (Emphasis added.)

11 In their paper on simulating clinical trials through Monte Carlo, Morris, White, and Crowther (2019) provide helpful analytic formula for deriving Monte Carlo standard errors for several diagnosands (“performance measures”). In the companion software, we adopt a non-parametric bootstrap approach that is able to calculate standard errors for any user-provided diagnosand.

12 This procedure depends on the researcher choosing a “good” diagnosand estimator. In nearly all cases, diagnosands will be features of the distribution of adiagnostic statistic that, given i.i.d. sampling, can be consistently estimated via plug-in estimation (for example taking sample means). Our simulation procedure, by construction, yields i.i.d. draws of the diagnostic statistic.

13 See also Collier, Brady, and Seawright (2004), Mahoney (2012), Bennett and Checkel (2014), Fairfield (2013).

14 For both methods, we use the “parsimonious” solution and not the “conservative” or“ intermediate” solutions that have been criticized in Baumgartner and Thiem (2017), though our declaration could easily be modified to check the performance of these alternative solutions.

15 Analternative might be to imagine a marginal effect conditional on actual assignment: if *x*_*i*_ is the observed treatment received by unit *i*, define, for small *δ*, $\tau \equiv E\left[Y_{i}\left(x_{i}\right)-Y_{i}\left(x_{i}-\delta \right)\right]/\delta$.

16 For instance researchers might be interested in a “conditional expectation function,” or in locating a parameter vector that can render a model as good as possible—such as minimizing the Kullback-Leibler information criterion (White 1982).

17 For a discussion of the distinctions between these different modes of replication, see Clemens (2017).

18 Of course, we could define our estimand as the difference in average weights for any surviving children in either state of the world: *E* [Weight(*Z* = 1)|Alive(*Z* = 1) = 1] − *E*[Weight(*Z* = 0)|Alive(*Z* = 0) = 1].This estimand would lead to very aberrant conclusions. Suppose, for example, that only one child with a very healthy weight survived in the control and all children, with weights ranging from healthy to very unhealthy, survived in the treatment. Despite all those lives saved, this estimand would suggest that the treatment has a large negative impact on health.

19 Raffler, Posner, and Parkerson (2019) employ a factorial design which breaks down the original intervention into two subcomponents: interface meetings between the community and village health teams, on the one hand, and integration of report cards into the action plans of health centers, on the other. We augment the sample size here only by the number of clusters corresponding to the pure control and both-arm conditions, as the other conditions of the factorial were not included in the original design. Including those other 189 clusters would only strengthen the conclusions drawn.

## APPENDIX

In this appendix, we demonstrate how each diagnosand-relevant feature of a simple design can be defined in code, with an application in which the assignment procedure is known, as in an experimental or quasi-experimental design.

*M*(1) **The population**. Defines the population variables, including both observed and unobserved *X*. In the example below we define a function that returns a normally distributed variable of a given size. Critically, the declaration is not a declaration of a particular realization of data but of a data generating *process.* Researchers will typically have a sense of the distribution of covariates from previous work, and may even have an existing data set of the units that will be in the study with background characteristics. Researchers should assess the sensitivity of their diagnosands to different assumptions about P(U).

population <-
  declare_population (N = 1000, u = rnorm
(N))

*M*(2) **The structural equations, or potential outcomes function**. The potential outcomes function defines conjectured potential outcomes given interventions *Z* and parents. In the example below the potential outcomes function maps from a treatment condition vector (*Z*) and background data *u*, generated by P(U) to a vector of outcomes. In this example the potential outcomes function satisfies a SUTVA condition-each unit’s outcome depends on its own condition only, though in general since *Z* is a vector, it need not.

potential_outcomes <-
  declare_potential_outcomes(Y~ 0.25 * Z
+ u)

In many cases, the potential outcomes function (or its features) is the very thing that the study sets out to learn, so it can seem odd to assume features of it. We suggest two approaches to developing potential outcomes functions that will yield useful information about the quality of designs. First, consider a null potential outcomes function in which the variables of interest are set to have no effect on the outcome whatsoever. Diagnosands such as bias can then be assessed relative to a true estimand of zero. This approach will not work for diagnosands like power or the Type-S rate. Second, set a series of potential outcomes functions that correspond to competing theories. This approach enables the researcher to judge whether the design yields answers that help adjudicate between the theories.

*I* **Estimands**. The estimand function creates a summary of potential outcomes. In principle, the estimand function can also take realizations of assignments as arguments, in order to calculate post-treatment estimands. Below, the estimand is the Average Treatment Effect, or the average difference between treated and untreated potential outcomes.

estimand <–
  declare_estimand(ATE = mean(Y_Z_1 –
Y_Z_0) )

*D*(1) **The sampling strategy**. Defines the distribution over possible samples for which outcomes are measured, *p*_*S*_.

In the example below each unit generated by P(U) is sampled with 10% probability. Again sampling describes a sampling strategy and not an actual sample.

sampling <– declare_sampling(n = 100)

*D*(2) **The treatment assignment strategy**. Defines the strategy for assigning variables under the notional control of researchers. In this example each sampled unit is assigned to treatment independently with probability 0.5. In designs in which the sampling process or the assignment process are in the control of researchers, *p*_*z*_ is known. In observational designs, researchers either know or assume *p*_*z*_ based on substantive knowledge. We make explicit here an additional step in which the outcome for *Y* is revealed after *Z* is determined.

assignment <– declare_assignment (m = 50)
reveal <– declare_reveal(Y, Z)

*A* **The answer strategies** are functions that use information from realized data and the design, but do not have access to the full schedule of potential outcomes. In the declaration we associate estimators with estimands and we record a set of summary statistics that are required to compute diagnostic statistics. In the example below, an estimator function takes data and returns an estimate of a treatment effect using the difference-in-means estimator, as well as a set of associated statistics, including the standard error, p-value, and the confidence interval.

estimator <– declare_estimator (Y ~ Z,
  model = lm_robust, estimand = “ATE”)

We then declare the design by adding together the elements. Order matters. Since we have defined the estimand before the sampling step, our estimand is the Population Average Treatment Effect, not the Sample Average Treatment Effect. We have also included a declare_reveal() step between the assignment and estimation steps that reveals the outcome Y on the basis of the potential outcomes and a realized random assignment.

design <–
  population + potential_outcomes +
estimand +
  sampling + assignment + reveal +
estimator

These six features represent the study. In order to assess the completeness of a declaration and to learn about the properties of the study, we also define functions for the diagnostic statistics, *t(D, Y,f),* anddiagnosands, *θ*(*D, Y*, *f,* g). For simplicity, the two can be coded as a single function. For example, to calculate the bias of the design as a diagnosand is:

diagnosand <– declare_diagnosands
  (bias = mean(estimate − estimand))

Diagnosing the design involves simulating the design many times, then calculating the value of the diagnosand from the resulting simulations.

diagnosis <–
  diagnose_design (design = design,
  diagnosands = diagnosand,
  sims = 500, bootstrap_sims = FALSE)

The diagnosis returns an estimate of the diagnosands, along with other metadata associated with the simulations.
