# Supplementary material for: Declaring and Diagnosing Research Designs
Source: Am Polit Sci Rev. Author manuscript; Available in PMC 2020 Aug 26. (PMC7449569; doi:10.1017/s0003055419000194)
Supplement: Supplemental Materials [file NIHMS1068676-supplement-Supplemental_Materials.pdf]

Supplementary Materials for ‘Declaring and Diagnosing Research Designs’

Graeme Blair, Jasper Cooper, Alexander Coppock, and Macartan Humphreys

Contents

1 Declaration of Simple Design **2**

2 Further details on survey of design tools **4**
2.1 Working Example 4
2.2 Search Method 5
2.3 Admissability Criteria 6
2.4 Coding Rules 6

3 Designs from section “Declaring and Diagnosing Research Designs in Practice” **8**
3.1 Descriptive Inference 8
3.1.1 Survey Designs 8
3.1.2 Bayesian Descriptive Inference 10
3.2 Causal Inference 12
3.2.1 Process Tracing 12
3.2.2 Qualitative Comparative Analysis 17
3.2.3 Nested Mixed Methods 23
3.2.4 Observational Regression-Based Strategies 30
3.2.5 Matching on Observables 32
3.2.6 Regression Discontinuity 34
3.2.7 Experimental Design 36
3.3 Designs for Discovery Research 38

4 Bjorkman and Svensson (2009) Design Replication **41**
4.1 Model 41
4.2 Inquiry 42
4.3 Data Strategy 43
4.4 Answer Strategy 43
4.5 Diagnosis of original design 44
4.6 Increasing Sample Size 45
4.7 Adding Covariates 46

References **49**

1 Declaration of Simple Design

The design discussed in Section "Elements of a Research Design" of the text can be formally declared and defined with the following code:

```
b <- 1

# Here we define a potential outcomes function
f_Y <- function(X, Z, b, e_y) b * X + Z + e_y

# Model -----------------------------------------------------------------
# Observed and unobserved variables; functional relations between them
model <- declare_population(
  N = 1000,
  e_y = rnorm(N),
  e_x = rnorm(N),
  Z   = rnorm(N),
  X   = Z   + e_x,
  Y   = f_Y(X, Z, b, e_y)
)

# Inquiry ---------------------------------------------------------------
# The average difference in the potential outcomes function
# evaluated at two points (X and X - 1)
inquiry <- declare_estimand(
  effect = mean(f_Y(X, Z, b, e_y) - f_Y(X - 1, Z, b, e_y)))

# Data Strategy ---------------------------------------------------------
# Sample n = 100 units
data_strategy <- declare_sampling(n = 100)

# Answer Strategy -------------------------------------------------------
# OLS regression of Y on X
answer_strategy <- declare_estimator(
  Y ~ X, model = lm_robust, estimand = "effect")

design <- model + inquiry + data_strategy + answer_strategy
```

```
if(do_diagnosis){
  diagnosis <-
    diagnose_design(
      design,
      diagnosands = declare_diagnosands(select = c(rmse, bias, mean_estimand)),
      sims = sims, bootstrap_sims = b_sims)
}
```

Estimand Label	Estimator Label	Term	N Sims	RMSE	Bias	Mean Estimand
effect	estimator	X	5000	0.51<br>(0.00)	0.50<br>(0.00)	1.00<br>(0.00)

With a design defined, it is relatively easy to alter it and assess results. For instance the below alters the design to one with a nonlinear data generating process. The estimand cannot be read from the dgp directly here but it still calculable.

```
# Alternative potential outcomes function
f_Y <- function(X, Z, b, e_y)  b*X^2 + e_y

design <- redesign(design, f_Y = f_Y)
```

In this new design there is no confounding, but diagnosis would reveal that there is still bias for this estimand

resulting from the non linearity of Y in X.

2 Further details on survey of design tools

This section describes the construction of the working example used in the research design tool survey, as well as the method used to search for tools to include in the survey, the criteria by which tools were admitted for inclusion into the survey, and the rules for coding the outcomes of this survey. In the online appendix we provide the raw data from the survey, including an overview of the tools considered for inclusion and the reasons for their eventual exclusion, as well as an archive of screenshots of all of the tools included in the survey itself. The tool survey was completed in July 2017 and all findings pertain to the tools we were able to locate at by that time point using the search methods described below.

2.1 Working Example

There are 1000 city blocks to choose from, each of which contains exactly 25 or 50 households, with the j'th block size distributed categorically, $n_j \sim \text{Cat}(\{25, 50\}, \{.5, .5\})$. Thus, the size of the sample varies as a function of which five city blocks the researcher randomly samples. Specifically, the expected sample size of the study is $N = 5 \times E[n] = 5 \times 37.5 = 187.5$.

Denoting the treatment variable $Z \in \{0, 1\}$, the i'th household respondent's potential outcomes are determined by the following system of equations

$$y_i = Z_i \alpha_j + \epsilon_i, \qquad (1)$$

with

$$\alpha_j \sim \text{N}\left(\frac{n_j}{100}, .1\right) \qquad Z_i \sim \text{Bin}\left(\frac{10}{n_j}\right) \qquad \epsilon_i \sim \text{N}(0, 1). \qquad (2)$$

Note that the size of the block determines respondents' potential outcomes and their probability of assignment to treatment. Specifically, the two are negatively correlated: the larger the respondent's block, the higher her treated potential outcome and the lower her probability of being assigned to the intervention.

The research design is declared and diagnosed using the following code:

```
design <-

  # Model -------------------------------------------------------------------

  # The data are heirarchical: individuals within blocks
  declare_population(
    block = add_level(N = 1000,
                      block_size = sample(c(25, 50), N, TRUE),
                      block_effect = rnorm(N, block_size / 100, .1)),
    individual = add_level(N = block_size,
                           noise = rnorm(N))) +

  # effects are different for different blocks
  declare_potential_outcomes(formula = Y ~ block_effect * Z + noise) +

  # Data Strategy -----------------------------------------------------------

  # sample n = 5 clusters of units
  declare_sampling(clusters = block, n = 5) +

  # treat m = 10 units per block
  declare_assignment(blocks = block, m = 10) +

  declare_reveal(Y, Z) +
```

```
# Inquiry --------------------------------------------------------------------

declare_estimand(ATE = mean(Y_Z_1 - Y_Z_0)) +

# Answer Strategy ------------------------------------------------------------

# three estimators
declare_estimator(Y ~ Z, model = lm_robust, se_type = "classical", label = "DIM", estimand = "ATE") +

declare_estimator(Y ~ Z + block, model = lm_robust, se_type = "classical", label = "BFE", estimand =

declare_estimator(Y ~ Z + block, weights = 1 / Z_cond_prob, model = lm_robust, se_type = "classical",
                  label = "IPW-BFE", estimand = "ATE")
```

This code produces the following diagnosis of the design:

```
if(do_diagnosis){
  diagnosis <-
    diagnose_design(
      design,
      sims = sims, bootstrap_sims = b_sims)
}
```

Table 2: Bias, RMSE, power and coverage of design in working example.

Design Label	Estimand Label	Estimator Label	Term	N Sims	Power	Bias	RMSE	Coverage
design	ATE	BFE	Z	5000	0.61<br>(0.01)	-0.02<br>(0.00)	0.17<br>(0.00)	0.94<br>(0.00)
design	ATE	DIM	Z	5000	0.60<br>(0.01)	-0.04<br>(0.00)	0.17<br>(0.00)	0.94<br>(0.00)
design	ATE	IPW-BFE	Z	5000	0.72<br>(0.01)	-0.00<br>(0.00)	0.18<br>(0.00)	0.90<br>(0.00)

The table above illustrates that the DIM and BFE estimators are negatively biased: they tend to underestimate the actual size of the treatment effect. This is because it is rarer for units with high treated potential outcomes to be assigned to treatment, a feature of the design that is not taken into account at all by the DIM estimator, and only through the estimation of a difference in intercepts by the BFE estimator. The IPW-BFE estimator has bias much closer to 0 because it reweights the data to take account of the lower probability with which units in larger blocks are assigned to treatment.

However, the IPW-BFE does not perform strictly better than the BFE estimator in this case. While its power is much higher, the coverage is below the nominal level of 0.95, indicating that the standard error estimator is anticonservative. For all three models, we estimated classical standard errors which rely on an additional modeling assumption of homoskedasticity. We could instead have estimated HC2 robust standard errors (by setting `se_type` to `"HC2"`) to address this problem.

In the following sections, we describe the methods by which we sought to assess the ability of available research tools to diagnose these features of the working example design.

2.2 Search Method

The survey sought to identify computational tools to diagnose the power and bias of the working example design described above. In terms of the identification criteria, we considered any software that promised to design and diagnose prospective research as a candidate for the survey.

We used two principle methods to search for candidates. First, we entered the search terms “statistical bias calculator” , “statistical power calculator” and “sample size calculator” into the Google web search engine,

using an incognito browser window in Google Chrome. We assessed the first 30 results using these terms. Second, we assessed the tools listed in four reviews of the literature, namely: Kreidler et al. (2013); Guo et al. (2013); Groemping (2016); Green and MacLeod (2016).

Using these two methods, we identified 143 candidate tools.

Since conducting the survey, the R package PowerUpR was released. We do not include the study here, nor do we include any tool that our search method was able to identify in July 2017. It is thus possible that we missed tools in our original survey. In the case of PowerUpR, while the package could handle power calculations for blocked random assignment as of August 10 2018, it could not incporporate heterogeneous block sizes or assignment probabilities, and thus likely exhibits the same shortcomings described with reference to the other tools surveyed.

2.3 Admissability Criteria

From the 143 candidate tools, we admitted 30 into the survey. We only admitted those tools that were specifically promised to calculate power or bias in a general purpose way, or in a way that was tailored to the working example. In other words, we excluded tools that were able to calculate power or bias but only for very specific designs that could not accommodate the working example. For instance, the `R` package `ThreeArmedTrials` was a candidate for inclusion because it was listed in the literature review by Groemping (2016) and promised to calculate power of experimental designs. However, because the tool was specifically set up to calculate the power of clinical non-inferiority or superiority trials, we excluded it from consideration in the survey. We also excluded research tools that serve to design research but are not set up to diagnose power or bias. For example, the `experiment` package is set up to design and analyze treatment effects in randomized experiments, but does not provide means for calculating power or bias of designs.

2.4 Coding Rules

Tools that were included in the survey were coded according to what information on a design they employed to calculate diagnosands (principally bias and power). Some tools accommodated information on design aspects (i.e., block sizes) but did not use this information in the calculation of diagnosands. Tools were only coded as employing a given piece of information if it was included in the calculation of diagnosands.

- *Effect sizes:* When rounded to the third decimal place, the PATE is $\approx .406$ with a standard deviation of 1.01, producing a Cohen's d of approximately .4. Thus, when a tool asked for an effect size without specifying what kind of effect, we entered a value of .4. Sometimes tools require an expression of the effect size in terms of Cohen's f^2. Unlike Cohen's d, the calculation of the f^2 requires that effects be specified in the context of a multivariate regression, and is thus difficult to calculate *a priori*. To calculate the f^2 in this context, we use the companion software to generate 500 R^2 under the full (block FE + treatment) and restricted (block FE only) models, and take the average of the f^2. This is perhaps overly generous to the assessed tools, as the f^2 estimated in this way encodes important design information that the tools do not ask for (such as the assignment probabilities).
- *Heterogeneous block sizes:* 1 if tool allows user to specify that units are organized into groups of different sizes, 0 otherwise.
- *Effect sizes correlated with block sizes:* 1 if tool allows user to specify that effects are correlated with group size, 0 otherwise.
- *Non-constant variance control vs. treatment:* 1 if tool allows for different variances in treatment vs. control, 0 otherwise.
- *Estimand:* 1 if tool allows user to formally define estimand as the Population Average Treatment Effect, 0 otherwise.
- *Sampling strategy:* 1 if tool allows user to specify anything about the strategy via which units are selected from the population into the sample, 0 otherwise.

- *Assign m within blocks:* 1 if tool allows users to specify that exactly m units will be assigned to treatment in the j'th block, 0 otherwise.
- *Inverse-probability weights:* 1 if tool allows users to specify that observations will be weighted by the inverse of their conditional assignment probability during estimation of effects, 0 otherwise.
- *Block fixed-effects:* 1 if tool allows users to specify that a block-level fixed-effect will be estimated, 0 otherwise.
- *Covariate adjustment:* 1 if tool allows users to account for conditioning on covariates, 0 otherwise.
- *Power of DIM:* the estimated power of the difference-in-means estimator if the tool is able to estimate it, NA otherwise.
- *Power of BFE:* the estimated power of the block fixed-effects estimator if the tool is able to estimate it, NA otherwise.
- *Power of IPW-BFE:* the estimated power of the inverse probability-weighted block fixed-effects estimator if the tool is able to estimate it, NA otherwise.
- *Bias:* the estimated bias of any of the estimators if the tool is able to estimate it, NA otherwise.
- *Coverage:* the estimated coverage of any of the estimators if the tool is able to estimate it, NA otherwise.

3 Designs from section "Declaring and Diagnosing Research Designs in Practice"

The code examples can be downloaded from the internet and run using the free, open source statistical package `R` and the `DeclareDesign` software.

3.1 Descriptive Inference

3.1.1 Survey Designs

- *M Model*: N voters have a latent probability of voting that is realized as actual voting through a probit process. The probability of voting is positively correlated with support for the Democratic candidate. People report their *support* truthfully, but are more likely to turn out to vote than they reveal.
- *I Inquiry*: We wish to know the true support for the Democratic candidate given that the respondent actually votes.
- *D Data Strategy*: We randomly sample 500 respondents from the U.S. population.
- *A Answer Strategy*: We estimate the true support for the Democratic candidate among those who will vote by taking the mean of stated support for the Democratic candidate among those who indicate they are likely voters.

3.1.1.1 Declaration

```
survey_design <-

  # Model ------------------------------------------------------------------

  declare_population(
    N = 1000,
    latent_voting = rnorm(N),
    latent_HRC_support = .2 * latent_voting + rnorm(N) - .2,
    voter = rbinom(N, 1, prob = pnorm(latent_voting)),
    HRC_supporter = rbinom(N, 1, prob = pnorm(latent_HRC_support)),
    # Subjects tend to overreport their probability of voting
    likely_voter = rbinom(N, 1, prob = pnorm(latent_voting + 0.75))) +

  # Inquiry ----------------------------------------------------------------

  # we're interested in the population level of true Clinton support among voters
  declare_estimand(true_support = mean(HRC_supporter), subset = voter == 1) +

  # Data Strategy ----------------------------------------------------------

  # We sample n = 500 with equal probability
  declare_sampling(n = 500) +

  # Answer Strategy --------------------------------------------------------

  # This regression just estimates a single mean
  # The estimator helpfully returns a confidence interval too, which is the reason
  # to use regression to estimate a single mean
  declare_estimator(HRC_supporter ~ 1,
                    model = lm_robust,
                    subset = likely_voter == 1,
                    term = "(Intercept)",
                    estimand = "true_support")
```

3.1.1.2 Diagnosis

```
if(do_diagnosis) {
  diagnosis <-
    diagnose_design(
      survey_design,
      diagnosands = declare_diagnosands(select = bias),
      sims = sims,
      bootstrap_sims = b_sims
    )
}
```

Design Label	Estimand Label	Bias
survey_design	true_support	-0.01
		(0.00)

3.1.2 Bayesian Descriptive Inference

- *M Model*: We posit a population of successes and failures generated through a probit process.
- *I Inquiry*: We wish to know the true probability of success.
- *D Data Strategy*: We sample 10 units.
- *A Answer Strategy*: We estimate empirical priors and a posterior distribution using a beta-binomial model. We compare two estimators, one that uses flat priors, and another that uses priors whose probability mass is centered at .5.

3.1.2.1 Declaration

```
# Model ------------------------------------------------------------------
population <- declare_population(N = 1000,
                                 noise = rnorm(N, -.1, .05),
                                 prob_success = pnorm(noise),
                                 success = rbinom(N, 1, prob_success))

# Inquiry ----------------------------------------------------------------
estimand <- declare_estimand(success_probability = mean(prob_success))

# Data Strategy ----------------------------------------------------------
sampling <- declare_sampling(n = 10)

# Answer Strategy --------------------------------------------------------

# here is a helper function that will be called by both estimators
beta_binom <-
  function(data, alpha_0, beta_0) {

    n_successes <- with(data, sum(success))

    n_trials <- nrow(data)

    alpha <- n_successes + alpha_0 - 1

    beta <- n_trials - n_successes + beta_0 - 1

    post <- dbeta(seq(0, 1, 0.005), alpha, beta)

    return(data.frame(
      post_mean = alpha / (alpha + beta),
      prior_sd = sqrt((alpha_0 * beta_0) /
                    (((alpha_0 + beta_0) ^ 2) * (alpha_0 + beta_0 + 1))),

      prior_mean = alpha_0 / (alpha_0 + beta_0),
      post_sd = sqrt((alpha * beta) /
                   (((alpha + beta) ^ 2) * (alpha + beta + 1)))))
  }

# an estimator with flat priors
estimator_flat_priors <-
  declare_estimator(handler = tidy_estimator(beta_binom),
                    alpha_0 = 1,
                    beta_0 = 1,
                    estimand = "success_probability",
                    label = "flat priors")

# only thing different here is the informative priors
estimator_info_priors <-
```

```
  declare_estimator(handler = tidy_estimator(beta_binom),
                    alpha_0 = 10,
                    beta_0 = 10,
                    estimand = "success_probability",
                    label = "informative priors")
# Design
bayesian_design <-
  population + estimand + sampling + estimator_flat_priors + estimator_info_priors
```

3.1.2.2 Diagnosis

```
# Some of these diagnosands are specific to Bayesian estimation strategies
bayesian_diagnosands <-
  declare_diagnosands(
    mean_est = mean(post_mean),
    mean_sd = mean(post_sd),
    bias = mean(post_mean - estimand),
    mean_shift = mean(post_mean - prior_mean),
    sd_shift = mean(post_sd - prior_sd),
    keep_defaults = FALSE)

# Two designs: n = 10 and n = 100.
bayesian_designs <- redesign(bayesian_design, n = c(10, 100))

if(do_diagnosis){
  diagnosis <- diagnose_design(
  bayesian_designs,
  diagnosands = bayesian_diagnosands,
  sims = sims, bootstrap_sims = FALSE)
}
```

n	Estimator Label	Mean Est	Mean Sd	Bias	Mean Shift	SD Shift
10	flat priors	0.46	0.14	0.00	-0.04	-0.15
10	informative priors	0.49	0.09	0.03	-0.01	-0.02
100	flat priors	0.46	0.14	0.00	-0.04	-0.15
100	informative priors	0.49	0.09	0.03	-0.01	-0.02

3.2 Causal Inference

3.2.1 Process Tracing

Our process tracing example draws upon the formalizations provided in Humphreys and Jacobs (2015) and Fairfield and Charman (2017).

- *M Model*: We posit a population of 195 cases, each of which does or does not exhibit the presence of an outcome, $Y \in \{0, 1\}$. For the sake of illustration, we will suppose that Y represents the presence or absence of a civil war. Each case also exhibits the presence or absence of a potential cause, $X \in \{0, 1\}$. For example, we might suppose that X represents the presence or absence of natural resources. In our posited model of the world, we specify that $Pr(X = 1) = 0.3$ for all cases: i.e., 30% of countries have natural resources.

The outcome Y can be realized through four distinct causal relations. First, the presence of X might cause Y, implying that for such cases: if $X = 0$, then $Y = 0$ and if $X = 1$ then $Y = 1$. In other words, civil wars happen in such cases *because* the country has natural resources. Second, the absence of X might cause Y: if $X = 0$ then $Y = 1$ and if $X = 1$ then $Y = 0$ in such cases. In such cases, civil war breaks out *because* the country does not have natural resources, and would not break out if the country had natural resources. Finally, Y might be present irrespective of X or Y might be absent irrespective of X. Continuing our analogy, such countries would have had civil war or peace, irrespective of whether they also had natural resources. We specify a model in which civil war is governed by causal pathway 1 (X causes Y) in roughly 20% of countries, by pathway 2 ($\neg X$ causes Y) in only 10% of countries, by pathway 3 (Y irrespective of X) in 20% of countries, and by pathway 4 ($\neg Y$ irrespective of X) in half of all countries.

- *I Inquiry*: We wish to know the answer to a "cause of effects" question. Specifically, we wish to know whether a specific case was one in which the presence (absence) of X caused the presence (absence) of Y: did civil war occur in this country because it had natural resources? Denoting the causal hypothesis that the presence of X causes the presence of Y by $H_{X \to Y}$, we denote our inquiry formally as $Pr(H_{X \to Y})$.
- *D Data Strategy*: We restrict our attention only to those cases in which both X and Y are present, and select one at random. In other words, we select only those cases in which civil war occurred and natural resources were present. By definition, we know that $Pr(H_{X \to Y} \mid X \neq Y) = 0$, for example, because $H_{X \to Y}$ is inconsistent with a data-generating process in which $X = 0, Y = 1$ or $X = 1, Y = 0$. It cannot be that natural resources were the cause of a civil war in a country that had a civil war but no natural resources. What we want to know is whether we see $Y = 1, X = 1$ *because* X caused Y. The inferential challenge is thus to discover whether the true reason that Y is present is because X caused it to be so.

The data strategy is to generate evidence in favor of one or another underlying causal process through the use of causal process observations (CPO) tests. In other words, if a country had a civil war because natural resources caused the civil war, there should be observable clues consistent with this hypothesis. The researcher specifies two CPO tests. The first is a "straw-in-the-wind": if X did not cause Y the researcher still expects to observe this CPO with probability 0.25, and if X did in fact cause Y the probability of observing the CPO is 0.75. The second is a "smoking-gun": this CPO is believed to arise with probability 0.05 if X is not the cause of Y, and with probability 0.30 if X is the cause of Y. Thus, the smoking-gun provides rare but definitive proof of the underlying causal process. In contrast, observing the straw-in-the-wind is more likely when the hypothesis that X caused Y is true, but can also happen when this hypothesis is false. For example, if, just prior to the civil war, an armed group was created whose main name, aims, and ideology were centered around the capture and control natural resources, this CPO may constitute a smoking gun. It is extremely unlikely to happen if $H_{X \to Y}$ is false, but might not happen even if it is true. The national army taking control over natural resources during a civil war is a straw-in-the-wind. This is very likely to happen if the natural resources caused the war, but also somewhat likely even if they did not. Finally, in addition to specifying beliefs about observing the CPOs depending on whether the hypothesis is true or false, the researcher also (implicitly) specifies a belief about the joint probability of observing both CPOs when the hypothesis that X caused Y is true or false. Namely, they specify that the CPOs are independent conditional on the hypothesis being true or false. In terms of our analogy, this is equivalent to assuming that, while

it is more likely to observe the argmed group and the national army's takeover of natural resources when resources truly did cause the civil war, this does not imply anything about the probability of observing the national army takeover *given that* the armed group was created. We relax this assumption below and show that it has strong and underexplored implications for process tracing inferences.

- *A Answer Strategy*: The researcher uses the CPOs in combination with Bayes' rule to update about the probability that X caused Y. In other words, they form a posterior inference, $Pr(H_{X \to Y} \mid E)$, where E denotes the CPOs they observe. We specify answer strategies for forming this inference. The first simply ignores the CPOs and is equivalent to stating a prior belief without doing any causal process tracing. The second looks only for a straw-in-the-wind, and the third looks only for a smoking-gun. These single-CPO strategies formalize the notion that process-tracing is time-consuming and costly. However, the fourth strategy conditions posterior inferences on both the straw-in-the-wind *and* the smoking-gun, which is consistent with the multiple-CPO strategies of many process-tracing applications (see, for example, Fairfield 2013).

3.2.1.1 Declaration

Here we declare the procedure described above in code. First, we'll declare a set of helper functions.

```
# Probability of observing straw-in-wind
# when H is TRUE vs. FALSE
pr_SIW_H <- .75
pr_SIW_not_H <- .25
# Probability of observing smoking gun
# when H is TRUE vs. FALSE
pr_SMG_H <- .30
pr_SMG_not_H <- .05
# Correlation in clues
rho_H <- 0
rho_not_H <- 0

# Calculate posterior given evidence
calculate_posterior <- function(data, p_H, p_clue_found_H, p_clue_found_not_H,
                                test, label) {
  clue_found <- data[, test]
  p_E_H <- ifelse(clue_found, p_clue_found_H, 1 - p_clue_found_H)
  p_E_not_H <- ifelse(clue_found, p_clue_found_not_H, 1 - p_clue_found_not_H)
  data.frame(posterior_H = p_E_H * p_H / (p_E_H * p_H + p_E_not_H * (1 - p_H)),
             clue_found = clue_found)}

# Calculate bivariate probabilities given correlation
joint_prob <- function(p1, p2, rho) {
  r <- rho * (p1 * p2 * (1 - p1) * (1 - p2)) ^ .5
  c(`00` = (1 - p1) * (1 - p2) + r,
    `01` = p2 * (1 - p1) - r,
    `10` = p1 * (1 - p2) - r,
    `11` = p1 * p2 + r)}

# Calculate posterior given correlated evidence
calculate_posterior_joint <- function(data, p_H, p_clue_1_found_H,
                                      p_clue_1_found_not_H, p_clue_2_found_H,
                                      p_clue_2_found_not_H, rho_H, rho_not_H,
                                      test){
  clue_found <- data[, test]
  p_E_H <- joint_prob(p1 = p_clue_1_found_H,
                      p2 = p_clue_2_found_H,
                      rho = rho_H)[clue_found]
  p_E_not_H <- joint_prob(p1 = p_clue_1_found_not_H,
                          p2 = p_clue_2_found_not_H,
                          rho = rho_not_H)[clue_found]
  data.frame(posterior_H = p_E_H * p_H / (p_E_H * p_H + p_E_not_H * (1 - p_H)),
```

```
              clue_found = clue_found)
}
```

The design itself makes use of the helper functions above in the `declare_estimator` steps.

```
process_tracing_design <-
  # Model -----------------------------------------------------------------

  declare_population(
    N = 195,
    X = rbinom(N, 1, .3) == 1,
    causal_process = sample(c('X_causes_Y', 'X_causes_not_Y', 'Y_regardless', 'not_Y_regardless'),
                            N, replace = TRUE, prob = c(.2, .1, .2, .5)),
    Y = (X & causal_process == "X_causes_Y") |
      (!X & causal_process == "X_causes_not_Y") |
      (causal_process == "Y_regardless"))  +

  # Data Strategy ---------------------------------------------------------
  declare_sampling(strata = (X == 1 & Y == 1),
                   strata_n = c("FALSE" = 0, "TRUE" = 1)) +

  # Inquiry ---------------------------------------------------------------
  # Sometimes Inquiries are defined *after* the data strategy.
  # Here the inquiry is defined after sampling.
  declare_estimand(did_X_cause_Y = causal_process == 'X_causes_Y') +

  # Answer Strategy -------------------------------------------------------
  declare_step(
    SIW_SMG = sample(c("00", "01", "10", "11"),1,
                     prob = {
                       if(causal_process == "X_causes_Y")
                         joint_prob(pr_SIW_H, pr_SMG_H, rho_H)
                       else
                         joint_prob(pr_SIW_not_H, pr_SMG_not_H, rho_not_H)
                     }),
  SIW_observed = SIW_SMG == "10" | SIW_SMG == "11",
  SMG_observed = SIW_SMG == "01" | SIW_SMG == "11",
  handler = fabricate,
  label = "Correlated Clues") +

  declare_estimator(
    test                 = "SIW_observed",
    p_H                  = .5,
    p_clue_found_H       = pr_SIW_H,
    p_clue_found_not_H = pr_SIW_not_H,
    label                = "Straw in Wind",
    estimand             = "did_X_cause_Y",
    handler              = tidy_estimator(calculate_posterior)) +

  declare_estimator(
    test                 = "SMG_observed",
    p_H                  = .5,
    p_clue_found_H       = pr_SMG_H,
    p_clue_found_not_H = pr_SMG_not_H,
    label                = "Smoking gun",
    estimand             = "did_X_cause_Y",
    handler              = tidy_estimator(calculate_posterior)) +

  declare_estimator(
    test                   = "SIW_SMG",
    p_H                    = .5,
    p_clue_1_found_H       = pr_SIW_H,
    p_clue_1_found_not_H = pr_SIW_not_H,
```

```
    p_clue_2_found_H     = pr_SMG_H,
    p_clue_2_found_not_H = pr_SMG_not_H,
    rho_H                = rho_H,
    rho_not_H            = rho_not_H,
    label                = "Update from both clues",
    estimand             = "did_X_cause_Y",
    handler              = tidy_estimator(calculate_posterior_joint))

# Diagnosands
process_tracing_diagnosands <-
  declare_diagnosands(
    bias = mean(posterior_H - estimand),
    rmse = sqrt(mean((posterior_H - estimand) ^ 2)),
    mean_estimand = mean(estimand),
    mean_posterior = mean(posterior_H),
    sd_posterior = sd(posterior_H),
    keep_defaults = FALSE
  )
```

3.2.1.2 Diagnosis

First, how do the different inferential strategies perform when we assume that the CPOs arise independently of one another, given the underlying causal process?

```
if(do_diagnosis) {
  diagnosis <-
    diagnose_design(
      process_tracing_design,
      diagnosands = process_tracing_diagnosands,
      sims = sims,
      bootstrap_sims = b_sims
    )
}
```

Estimator Label	Bias	RMSE	Mean Estimand	Mean Posterior	SD Posterior
Smoking gun	-0.00	0.47	0.51	0.50	0.17
	(0.01)	(0.00)	(0.01)	(0.00)	(0.00)
Straw in Wind	-0.00	0.44	0.51	0.50	0.25
	(0.01)	(0.00)	(0.01)	(0.00)	(0.00)
Update from both clues	-0.00	0.41	0.51	0.51	0.28
	(0.01)	(0.00)	(0.01)	(0.00)	(0.00)

Comparing between strategies where a researcher commits ex ante to only search for smoking guns or straws-in-the-wind, the results are somewhat surprising. First, as expected, the standard deviation in posterior inferences generated by the smoking gun strategy is much lower than that provided by the straw-in-the-wind, because the smoking gun provides greater certainty conditional on observing the CPO. However, on average the RMSE is lower when one only searches for straws-in-the-wind, because they are more commonly observed. By this criterion, the straw-in-the-wind strategy actually outperforms the smoking gun strategy. Clearly, however, RMSE is minimized by conditioning on both CPOs. But here we have specified that the CPOs provide independent information on the true underlying hypothesis: what about when the CPOs are correlated?

We diagnose the design for cases in which the tests are negatively correlated and positively correlated. Negatively correlated CPOs might arise if they result from substitute processes (either from one path or an alternative path). For example, if the national army is less likely to take control of the natural resources precisely when an armed group has declared that it will fight for them. Positively correlated CPOs might arise if they result from common processes (two observations that arise on the same path). For example, if the national army takes control over natural resources precisely because this counters the stated strategic objectives of the armed group.

```
process_tracing_designs <- redesign(process_tracing_design,
                                    rho_H = c(-0.32, 0, + 0.32))

if(do_diagnosis){
  diagnosis_2 <-
    diagnose_design(
      process_tracing_designs,
      diagnosands = process_tracing_diagnosands,
      sims = sims,
      bootstrap_sims = b_sims)
}
```

rho_H	Estimator Label	RMSE
-0.32	Update from both clues	0.39 (0.00)
0.32	Update from both clues	0.42 (0.00)

We see that if two CPOs are sought expected errors are lower when these are negatively correlated with each other. This feature arises because the CPO tests carry less independent information when they are positively correlated. To see this, suppose they were perfectly correlated, so that seeing one guaranteed the other would also be present. In this case, there is no additional information gleaned from the observation of one CPO once the other has been observed: they are effectively equivalent tests.

3.2.2 Qualitative Comparative Analysis

Our QCA example examines "Crisp Set" QCA in line with the formalizations provided in Ragin (1987) and drawing on the QCA package developed by Thiem and Dusa (2013). See also Duşa (2018).

- *M Model*: We suppose there exist N cases. Whether those cases exhibit an outcome, Y, is determined by the configuration of causal conditions that those cases feature. Specifically, the absence of A and the presence of B are necessary and sufficient causes of the presence of Y. We denote this relationship $Y = a * B$, where lower case a indicates the absence of A and upper case B indictates the presence of B. In the code below we use binary indicators for presence and absence, so that $A = 0$ is equivalent to a and $A = 1$ is equivalent to A, for example. The $*$ operator is equivalent to "and." The causal relationship can be read "Y happens if and only if A is absent and B is present."
- *I Inquiry*: We wish to know the true set of causal configurations that produce Y.
- *D Data Strategy*: We assume that the researcher does not have direct access to Y, but must encode presence or absence in a truth table. We allow for some error in this coding, but make no claim about what this error represents. For example, it may be that scholarly debate generates epistemic uncertainty about whether Y is truly present or absent in a given case, or that there is measurement error due to sampling variability (Rohlfing 2018).
- *A Answer Strategy*: We consider two answer strategies initially. The first employs the classical Quine-McCluskey minimization algorithm (see Duşa and Thiem 2015 for a definition) and the second the "Consistency Cubes" algorithm (Duşa 2018) to solve for the set of causal conditions that produces Y. Further below, we also consider how least squares minimization performs when targeting a QCA estimand. The righthandside of the regression includes indicators for membership in all feasible configurations of A and B. Configurations that yield predictions for Y greater than 0.5 are then included in the set of sufficient conditions.

3.2.2.1 Declaration

We start by declaring the sample size and assume that the outcome is never miscoded.

```
library(QCA)

N <- 6
error_rate <- 0
# Model ----------------------------------------------------------------
cases <-
  declare_population(N = N,
                     A = rbinom(N, 1, .2),
                     B = rbinom(N, 1, .8))

counterfactuals <-
  declare_potential_outcomes(Y ~ 1 * (A == 0 & B == 1),
                             conditions = list(A = 0:1, B = 0:1))

# Inquiry --------------------------------------------------------------
estimand <-
  declare_estimand(true_configuration = "a*B")

# Data Strategy --------------------------------------------------------

true_outcome <-
  declare_reveal(Y, assignment_variables = c(A, B))

code_outcome <-
  declare_step(
    mistake = rbinom(N, 1, error_rate) == 1,
    Y_obs = (1 - mistake) * Y + mistake * (1 - Y),
```

```r
    handler = fabricate
  )

# Answer Strategy ----------------------------------------------------------

# This function returns the output of QCA::minimize() in a tidy data.frame.
minimization_algorithm <-
  function(data, method, label) {
    estimate <- tryCatch(
      expr = {
        truth_table <-
          truthTable(
            data = data,
            outcome = "Y_Obs",
            incl.cut = 0.5,
            conditions = c("A", "B")
          )

        solutions <-
          minimize(truth_table, include = "?", method = method)$solution

        paste(unique(unlist(solutions)), collapse = " + ")
      },
      error = function(e) "NO SOLUTION"
    )
    return(
      data.frame(
        estimate = estimate,
        estimand_label = "true_configuration",
        estimator_label = label,
        stringsAsFactors = FALSE
      )
    )
  }

# The Quine-McCluskey algorithm
QMC_estimator <-
  declare_estimator(handler = minimization_algorithm,
                    method = "QMC",
                    label = "Classic Quine-McCluskey")

# The Consistency Cubes algorithm
CC_estimator <-
  declare_estimator(handler = minimization_algorithm,
                    method = "CCubes",
                    label = "Consistency Cubes")

QCA_design <-
  cases +
  counterfactuals +
  estimand +
  true_outcome +
  code_outcome +
  QMC_estimator +
  CC_estimator
```

One draw of the data looks as follows:

ID	A	B	Y_A_0_B_0	Y_A_1_B_0	Y_A_0_B_1	Y_A_1_B_1	Y	mistake	Y_obs
1	0	0	0	0	1	0	0	FALSE	0
2	1	1	0	0	1	0	0	FALSE	0
3	0	1	0	0	1	0	1	FALSE	1
4	1	1	0	0	1	0	0	FALSE	0

ID	A	B	Y_A_0_B_0	Y_A_1_B_0	Y_A_0_B_1	Y_A_1_B_1	Y	mistake	Y_obs
5	0	1	0	0	1	0	1	FALSE	1
6	0	1	0	0	1	0	1	FALSE	1

One draw of the estimates looks as follows:

estimate	estimand_label	estimator_label
B	true_configuration	Classic Quine-McCluskey
B	true_configuration	Consistency Cubes

In principle there is a broad range of interesting diagnosands to explore. These include notions of "coverage" and "inclusion" but also power (Rohlfing 2018). For simplicity we here focus on two. The first is the probability that our answer strategy produces the *exact* set of necessary and sufficient causal combinations that produce the outcome. The second is the probability that our answer strategy fails to provide a an answer at all (no solution to the minimization problem).

```
QCA_diagnosands <-
  declare_diagnosands(
    correct_rate = mean(estimand == estimate),
    failure_rate = mean(estimate == "NO SOLUTION"),
    keep_defaults = FALSE
  )
```

3.2.2.2 Diagnosis

```
if(do_diagnosis) {
  diagnosis <-
    diagnose_design(
      QCA_design,
      diagnosands = QCA_diagnosands,
      sims = sims,
      bootstrap_sims = b_sims
    )
}
```

Estimator Label	Correct Rate	Failure Rate
Classic Quine-McCluskey	0.39	0.07
	(0.01)	(0.00)
Consistency Cubes	0.39	0.00
	(0.01)	(0.00)

The first thing to note is that the two algorithms perform relatively similarly. The consistency cubes algorithm does return answers more often than the classic algorithm. However, those answers are not necessarily more accurate. Around 39% of the time, both classic QMC and CCubes return exactly the right causal configuration.

How do these diagnosands change as both the rate of miscoding errors and the sample size change? To investigate this, we can redesign and diagnose our original design.

```
QCA_designs <-
  redesign(QCA_design,
           error_rate = c(0, 0.05),
           N = c(5, 50))

if (do_diagnosis) {
  diagnosis_2 <-
```

```
    diagnose_design(
      QCA_designs,
      diagnosands = QCA_diagnosands,
      sims = sims,
      bootstrap_sims = b_sims
    )
}
```

Estimator Label	error_rate	N	Correct Rate	Failure Rate
Classic Quine-McCluskey	0	5	0.31 (0.01)	0.11 (0.00)
Consistency Cubes	0	5	0.31 (0.01)	0.01 (0.00)
Classic Quine-McCluskey	0.05	5	0.26 (0.01)	0.14 (0.00)
Consistency Cubes	0.05	5	0.26 (0.01)	0.01 (0.00)
Classic Quine-McCluskey	0	50	1.00 (0.00)	0.00 (0.00)
Consistency Cubes	0	50	1.00 (0.00)	0.00 (0.00)
Classic Quine-McCluskey	0.05	50	0.96 (0.00)	0.00 (0.00)
Consistency Cubes	0.05	50	0.96 (0.00)	0.00 (0.00)

Turning first to how the diagnosands vary by N, we see that both algorithms work perfectly when there is no miscoding of the outcome and the sample is large. Measurement error reduces the accuracy of the solutions in quantifiable ways – that is, a researcher can assess, for any given true data generating process, the probability with which measurement error will yield to a misidentification of causal paths.

How might the use of a simple linear minimization algorithm perform under the existence of miscoding errors? To investigate this, we declare an answer strategy that uses linear regression to find the configuration of causal conditions that produces Y.

```
# The linear minimization algorithm requires a separate helper function
linear_minimization <-
  function(data, label) {
    # Get coefficients from saturated model
    betas <- coef(lm(Y_obs ~ A * B, data = data))

    # Assign any dropped coefficients 0
    betas[is.na(betas)] <- 0

    # Linear model prediction of Pr(Y|A,B)
    predictions <- c(
      "a*b" = betas[1],
      "A*b" = betas[1] + betas[2],
      "a*B" = betas[1] + betas[3],
      "A*B" = betas[1] + betas[2] + betas[3] + betas[4]
    )

    # Grab all configurations that predict Pr(Y) > .5
    configurations <- c("a*b", "A*b", "a*B", "A*B")[predictions > 0.5]

    if (!any(predictions > 0.5)) {
      # If no configurations produce the outcome, no solution found
      estimate <- "NO SOLUTION"

    } else {
```

```
      # If configurations are found, translate implicants to canonical form
      sum_of_products <- try(QCA::simplify(paste(configurations, collapse = "+")), silent = TRUE)

      # If configurations are contradictory, no solution found
      estimate <- ifelse(class(sum_of_products) %in% "try-error", "NO SOLUTION", sum_of_products)
    }

    return(data.frame(
      estimate = estimate,
      estimand_label = "true_configuration",
      estimator_label = label))
  }

linear_estimator <-
  declare_estimator(handler = linear_minimization, label = "Least Squares")
```

We add the estimator to our original design, modify it to include combinations of a larger sample size and error rate as above, and diagnose the designs' properties. We declare a design in which $N = 3$ to highlight the point that linear regression is capable estimating more parameters than it has degrees of freedom—more generally, for estimation purposes small numbers of cases and great causal complexity do not preclude the use of regression approaches.

```
QCA_design_with_lm <- QCA_design + linear_estimator

QCA_designs_with_lm <- redesign(QCA_design_with_lm,
                                error_rate = c(0, 0.20), N = c(3, 50))
if(do_diagnosis){
  diagnosis_3 <-
    diagnose_design(
      QCA_designs_with_lm,
      diagnosands = QCA_diagnosands,
      sims = sims,
      bootstrap_sims = b_sims
    )
}
```

Estimator Label	error_rate	N	Correct Rate	Failure Rate
Classic Quine-McCluskey	0	3	0.10<br>(0.00)	0.32<br>(0.01)
Consistency Cubes	0	3	0.10<br>(0.00)	0.05<br>(0.00)
Least Squares	0	3	0.00<br>(0.00)	1.00<br>(0.00)
Classic Quine-McCluskey	0.2	3	0.05<br>(0.00)	0.45<br>(0.01)
Consistency Cubes	0.2	3	0.05<br>(0.00)	0.10<br>(0.00)
Least Squares	0.2	3	0.00<br>(0.00)	1.00<br>(0.00)
Classic Quine-McCluskey	0	50	1.00<br>(0.00)	0.00<br>(0.00)
Consistency Cubes	0	50	1.00<br>(0.00)	0.00<br>(0.00)
Least Squares	0	50	0.00<br>(0.00)	1.00<br>(0.00)
Classic Quine-McCluskey	0.2	50	0.72<br>(0.01)	0.00<br>(0.00)
Consistency Cubes	0.2	50	0.72<br>(0.01)	0.00<br>(0.00)
Least Squares	0.2	50	0.00<br>(0.00)	1.00<br>(0.00)

Interestingly, the three approaches yield almost exactly the same results when the sample size is small. The main difference resides in the case when the measurement error *and* sample size are large. In such cases, the saturated regression approach slightly outperforms the Boolean minimization algorithms. The diagnosis suggests that QCA can be fruitfully integrated with saturated regression approaches.

3.2.3 Nested Mixed Methods

We are interested in knowing the answer to an effect-of-causes question, here the effect of X on Y, though our strategy also examines causes-of-effects questions in order to help address it. We employ the nested case analysis strategy proposed in Lieberman (2005).

We build on this framework by examining the implications of several tradeoffs faced by researchers employing nested analysis. For example, is it better to invest effort into theory testing or into theory building? What are the consequences of being more or less lenient with regard to the rejection of large- and small-N analyses?

- *M Model*: We posit a model of the world in which X causes Y but the effect is confounded by a variable, W, that the researcher can learn about through qualitative small-N analysis.
- *I Inquiry*: We wish to know the true average effect of X on Y.
- *D Data Strategy* and *A Answer Strategy*: The data strategy and analysis strategy are intertwined. We start with a simple theory, namely that X has similar effects on Y for all units and that the effect of X on Y is unconfounded, and test it on our large-N dataset. The results of this analysis are deemed "satisfactory and robust" if the residual variance from a regression $Y \sim X$ falls below a given threshold, which is set by the researcher as part of the nested strategy. If the the regression is deemed satisfactory, we engage in small-N model-testing by selecting cases on the regression line (i.e., for which $X = 0, Y = 0$ or $X = 1, Y = 1$). We suppose that small-N qualitative analysis reveals whether Y was truly caused by X in the cases studied, but it is time-consuming and costly to do, so it is limited to a small number. If X caused Y in a satisfactory share of cases, which is determined by a threshold set by the researcher as part of their strategy, then the initial model is accepted. If the initial theory fails to explain an adequate share of cases – or if the initial large-N model was deemed to leave too much residual variance unexplained – we move to the theory-building small-N analysis. Here, we suppose that the researcher learns of the variable W, and updates to a new theory in which Y is a function of both X and W if the way that X affects Y is sufficiently different in the presence or absence of W. If there is sufficient evidence that the effect of X on Y is moderated by W, then it is included in a new updated theory. Finally, the researcher returns to the large-N analysis. If they have been led to update their model they use the new one, otherwise they forego answering their research question.

The simplified version of Figure 1 in Lieberman (2005) that we employ here is depicted below.

3.2.3.1 Declaration

First, we set the values of some parameters we will be using in the declaration.

```
# Effect of X on Y when W = 1
ate <- 0.50

# Confounding between X and W
rho <- 0.70

# Maximal residual variance for
# original LNA model to be deemed satisfactory
LNA_threshold <- 0.20

# Minimal share of causal claims that must be validated
# in mt-SNA for original theory to be deemed valid
SNA_threshold_1 <- 1/3

# Minimal size of difference in effect of X on Y for W
# to be deemed important enough to update theory
SNA_threshold_2 <- 0.10

# Amount of effort put into qualitative analysis
# for testing existing theory
# model_testing_effort = 1 implies 4 cases tested
```

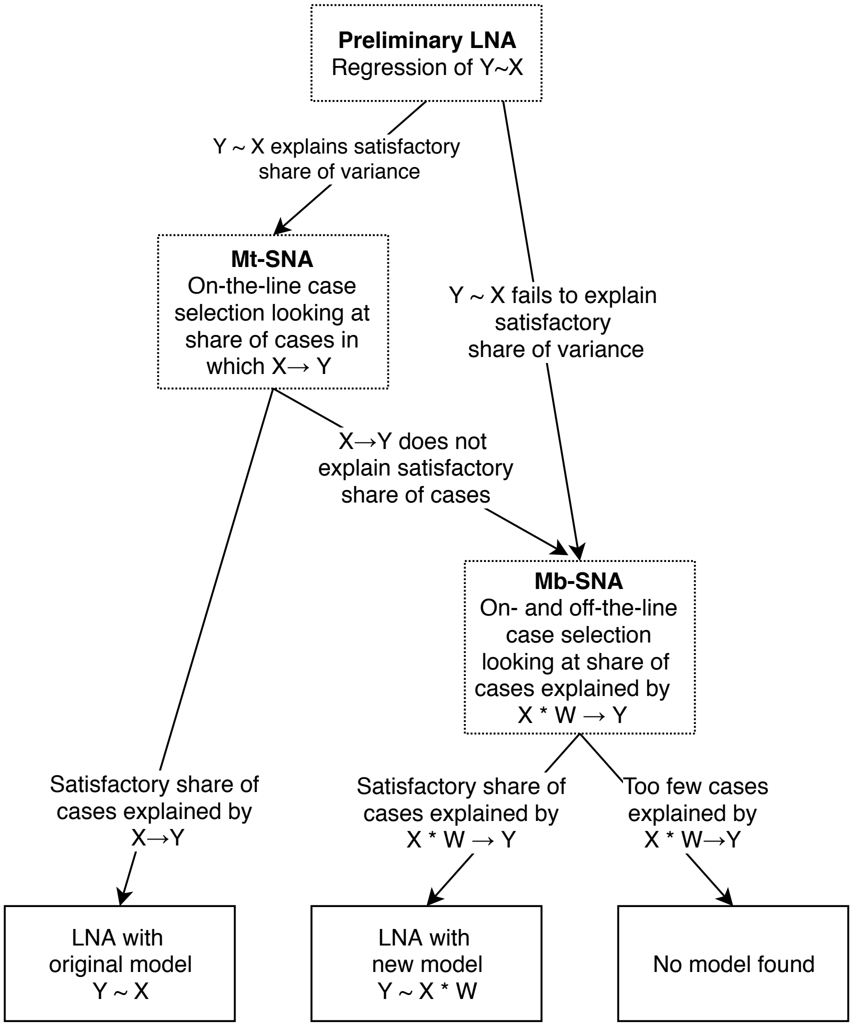

Figure 1: A simplified acyclic version of Lieberman (2005).

```
# model_testing_effort = 2 implies 8 cases tested
model_testing_effort <- 1

# Amount of effort put into qualitative analysis
# for building new theory
# model_building_effort = 1 implies W investigated in 4 cases
# model_building_effort = 2 implies W investigated in 8 cases
model_building_effort <- 1
```

The actual declaration begins here

```
# Model -----------------------------------------------------------------
# Model specifies that X causes Y, but confounded by W
LN_data <-
  declare_population(N = 1000,
                     W = rbinom(N, 1, prob = 0.5),
                     X = correlate(given = W, rho = rho, draw_binary, prob = 0.5),
                     u = runif(N),
                     Y_X_0 = W * (u > (1 + ate) / 2),
                     Y_X_1 = W * (u > (1 - ate) / 2),
                     Y = X * Y_X_1 + (1 - X) * Y_X_0,
                     outcome = paste0(X, Y))


# Inquiry ---------------------------------------------------------------
# We want to know the true average effect of X on Y
true_effect <- declare_estimand(X_on_Y = mean(Y_X_1 - Y_X_0))

# Data Strategy ---------------------------------------------------------
# We start with a simple model and see
# whether X explains enough of Y
LNA <-
  declare_step(
    LN_res_var = lm_robust(Y ~ X)$res_var,
    LNA_satisfactory = LN_res_var < LNA_threshold,
    handler = fabricate
  )

# Depending on what we find we will select
# only cases on the Y~X regression line

select_on_line <-
  declare_sampling(
      strata = outcome,
      strata_n = model_testing_effort * c("00" = 2, "01" = 0, "10" = 0, "11" = 2))

# ... or off and on the Y~X regression line
select_on_and_off_line <-
  declare_sampling(
    strata = outcome,
    strata_n = model_building_effort * c("00" = 1, "01" = 1, "10" = 1, "11" = 1))


# Answer Strategy -------------------------------------------------------

SNA <-
  declare_step(
      handler = function(data){

        LNA_satisfactory <- all(data$LNA_satisfactory)

        # First, is the LNA deemed satisfactory?
        if(LNA_satisfactory) {
```

```r
          # If so, select cases on the line
          SN_data <- select_on_line(data)
          # And determine whether a sufficient share exhibit the posited causal
          # relationship
          SNA_fits_LNA <- mean(with(SN_data,Y_X_1 - Y_X_0)) > SNA_threshold_1
        } else {
          SNA_fits_LNA <- FALSE
        }

        if(LNA_satisfactory & SNA_fits_LNA){
          # If both the LNA and SNA accord with respect to the first model
          # then accept it
          data$situation <- "I: Original Model Accepted"
        } else {
          # Otherwise go back to the full data and sample on and off the line
          SN_data <- select_on_and_off_line(data)
          # Look at effect of X and Y by whether W = 1 or W = 0
          Y_on_X_W <- with(subset(SN_data, W == 1), mean(Y_X_1 - Y_X_0))
          Y_on_X_no_W <- with(subset(SN_data, W == 0), mean(Y_X_1 - Y_X_0))
          # Determine whether the X->Y relationship is moderated by W
          evidence_for_W <- Y_on_X_no_W - Y_on_X_W
          evidence_for_W <- ifelse(is.na(evidence_for_W),0, abs(evidence_for_W))
          new_model_discovered <- evidence_for_W > SNA_threshold_2
          data$situation <- ifelse(test = new_model_discovered,
                                   # If W seems to moderate the X->Y
                                   # relationship, update the model
                                   yes = "II: New Model Accepted",
                                   # otherwise, give up
                                   no = "IV: Original Model Rejected, No New Model")
        }
        return(data)
      })

# For the final LNA, use the original model if it was kept
original_LNA <- declare_estimator(Y ~ X,
                                  model = lm_robust,
                                  estimand = true_effect,
                                  label = "Original Model")

# Otherwise use the new model, accounting for X*W interaction
new_LNA <-
  declare_estimator(
    Y ~ X,
    covariates = ~ W,
    model = lm_lin,
    estimand = true_effect,
    label = "New Model"
  )

final_analysis <-
  declare_estimator(
    handler = function(data) {
      situation <- unique(data$situation)
      if (situation == "I: Original Model Accepted")
        return(original_LNA(data))
      if (situation == "II: New Model Accepted")
        return(new_LNA(data))
      if (situation == "IV: Original Model Rejected, No New Model") {
        return(
          data.frame(
            estimator_label = "No Model",
            term = "X",
            estimate = NA,
```

```
            std.error = NA,
            statistic = NA,
            p.value = NA,
            conf.low = NA,
            conf.high = NA,
            df = NA,
            outcome = "Y",
            estimand_label = "X_on_Y"
          )
        )
      }
    }
  )

# Declare the design
nested_design <- LN_data + true_effect + LNA + SNA + final_analysis

nested_diagnosands <-
  declare_diagnosands(
    bias = mean(estimate - estimand, na.rm = TRUE),
    rmse = (mean((estimate - estimand) ^ 2, na.rm = TRUE)) ^ .5,
    mean_estimate = mean(estimate, na.rm = TRUE),
    mean_estimand = mean(estimand),
    keep_defaults = FALSE
  )
```

3.2.3.2 Diagnosis

We first analyze how confounding poses problems for inference.

```
confounding_designs <- redesign(nested_design, rho = c(0, 0.5))

if(do_diagnosis){
  diagnosis <-
    diagnose_design(
      confounding_designs,
      diagnosands = nested_diagnosands,
      sims = sims, bootstrap_sims = b_sims)
}
```

rho	Estimator Label	Bias	RMSE	Mean Estimate	Mean Estimand
0	New Model	0.00	0.02	0.25	0.25
		(0.00)	(0.00)	(0.00)	(0.00)
0	No Model	NaN	NaN	NaN	0.25
		NA	NA	NA	(0.00)
0	Original Model	-0.00	0.02	0.25	0.25
		(0.00)	(0.00)	(0.00)	(0.00)
0.5	New Model	0.00	0.02	0.25	0.25
		(0.00)	(0.00)	(0.00)	(0.00)
0.5	No Model	NaN	NaN	NaN	0.25
		NA	NA	NA	(0.00)
0.5	Original Model	0.15	0.15	0.40	0.25
		(0.00)	(0.00)	(0.00)	(0.00)

As one might expect, the original model is biased if there is truly confounding. Thus, the core question for our nested case strategy is what combination of thresholds and effort devoted to qualitative case analysis will maximize our chances of selecting the correct model?

We look first at how the amount of effort put into different qualitative strategies changes our probability of selecting the right theory of the $X \rightarrow Y$ relatonship. When `model_testing_effort = 1` and

`model_building_effort = 5`, that means we carry out small-N testing in four cases but do theory building in 20, for example.

```
sna_designs <-
  redesign(
    nested_design,
    model_testing_effort = c(5, 1),
    model_building_effort = c(1, 5)
  )

if(do_diagnosis){
  diagnosis_2 <-
    diagnose_design(
      sna_designs,
      diagnosands = nested_diagnosands,
      sims = sims, bootstrap_sims = b_sims)
}
```

The table below reveals a surprising feature of the nested analysis strategy: increasing the effort devoted to testing *decreases* our chances of happening on the right model. In fact, we are better off when more effort is devoted to qualitative theory building and less effort to qualitative theory testing. The exercise highlights the importance of theory-building.

model_testing_effort	model_building_effort	estimator_label	prop_sims
1	1	New Model	0.2780
1	5	New Model	0.2858
5	1	New Model	0.2782
5	5	New Model	0.2752

What can we say about how the weight we put on different forms of evidence affect the likelihood we arrive at the best model? We analyze eight different designs combining low and high thresholds for each of the different decision nodes in our nested strategy. At each, we look at `prop_sims`: the proportion of simulations of the design in which we arive at the correct model.

A low threshold on the LNA means we are less likely to accept an original large-N theory even if it explains a relatively high share of the variance in the outcome. A high threshold for the first SNA test implies we require a large share of cases to exhibit the posited causal relationship in our qualitative model-testing stage in order for us to declare it consistent with the LNA. Finally, a high threshold on the second SNA test implies that we require a large difference in effects in order to believe the alternative theory.

```
threshold_designs <-
  redesign(
    nested_design,
    LNA_threshold = c(0.1, 0.8),
    SNA_threshold_1 = c(0.1, 0.8),
    SNA_threshold_2 = c(0.05, 0.3)
  )

if(do_diagnosis){
  diagnosis_3 <-
    diagnose_design(
      threshold_designs,
      diagnosands = nested_diagnosands,
      sims = sims, bootstrap_sims = b_sims)
}
```

LNA_threshold	SNA_threshold_1	SNA_threshold_2	estimator_label	prop_sims
0.1	0.8	0.05	New Model	0.6424
0.1	0.8	0.30	New Model	0.6270
0.1	0.1	0.05	New Model	0.6244

LNA_threshold	SNA_threshold_1	SNA_threshold_2	estimator_label	prop_sims
0.1	0.1	0.30	New Model	0.6242
0.8	0.1	0.30	New Model	0.2840
0.8	0.8	0.30	New Model	0.2748
0.8	0.1	0.05	New Model	0.2740
0.8	0.8	0.05	New Model	0.2738

The results suggest that leniency with respect to SNA and LNA testing increase the probability of accepting the initial model, and therefore decrease the quality of inferences.

Of course, these insights do not necessarily generalize to alternative nested case analysis strategies, using different thresholds or assuming different inferential problems. However, the declaration shows that it is possible to declare and diagnose very complex inferential procedures whose tradeoffs are otherwise unclear.

3.2.4 Observational Regression-Based Strategies

- *M Model*: We posit a population whose potential outcomes are non-linearly but monotonically increasing in a variable, Z.
- *I Inquiry*: We wish to know the average change in potential outcomes brought about by increasing Z by one unit. We operationalize this in two ways: first by calculating a parameter for a linear effect from a linear model fitting outcomes in the space of potential outcomes; second as the average effect of a change in treatment, calculated using the potential outcomes directly.
- *D Data Strategy*: We imagine two processes through which the values of Z are assigned: in the first, each value is assigned with equal probability; in the second, the highest value is assigned with a lower probability than the lower values.
- *A Answer Strategy*: We estimate the effect of an average unit increase in Z through linear regression of the outcome on Z.

3.2.4.1 Declaration

Some parameters we will use in the design:

```
f_Y <- function(Z, u) Z ^ .5 + u    # A conjectured data generating process

model_estimand_function <-  function(Y_Z_1, Y_Z_2, Y_Z_3) {
  YY <- c(Y_Z_1, Y_Z_2, Y_Z_3)
  XX <- rep(1:3, each = length(Y_Z_1))
  coef(lm(YY ~ XX))[2]}

 prob_each <- c(1, 1, 1) / 3    # Assignment probabilities: we will modify these
```

The design:

```
model_estimand <-

# Model -----------------------------------------------------------------
  declare_population(N = 10, u = rnorm(N)) +
  declare_potential_outcomes(Y ~ f_Y(Z, u), conditions = 1:3) +

# Inquiry ---------------------------------------------------------------
  declare_estimand(
    beta = model_estimand_function(Y_Z_1, Y_Z_2, Y_Z_3),
    aate = mean((f_Y(3, u) - f_Y(1, u))/2)) +

# Data Strategy ---------------------------------------------------------
  declare_assignment(conditions = 1:3, prob_each = prob_each) +
  declare_reveal(Y, Z) +

# Answer Strategy -------------------------------------------------------
  declare_estimator(Y ~ Z, model = lm_robust,
                    estimand = c("beta", "aate"), label = "ols")
```

3.2.4.2 Diagnosis

We diagnose the two versions of the design with equal and unequal assignment probabilities.

```
designs <- redesign(model_estimand, prob_each = list(c(1,1,1)/3, c(.4, .4, .2)))

if(do_diagnosis){
  diagnosis <-
    diagnose_design(
      designs,
```

```
      sims = sims, bootstrap_sims = b_sims)
}
```

Design Label	Mean Estimand	Estimand Label	prob_each	Bias	RMSE	Power	Coverage
design_1	0.37<br>(0.00)	aate	c(1, 1, 1)/3	0.00<br>(0.01)	0.39<br>(0.00)	0.16<br>(0.01)	0.93<br>(0.00)
design_1	0.37<br>(0.00)	beta	c(1, 1, 1)/3	0.00<br>(0.01)	0.39<br>(0.00)	0.16<br>(0.01)	0.93<br>(0.00)
design_2	0.37<br>(0.00)	aate	c(0.4, 0.4, 0.2)	0.00<br>(0.01)	0.42<br>(0.00)	0.15<br>(0.01)	0.93<br>(0.00)
design_2	0.37<br>(0.00)	beta	c(0.4, 0.4, 0.2)	0.00<br>(0.01)	0.42<br>(0.00)	0.15<br>(0.01)	0.93<br>(0.00)

We confirm first that the two linear estimands are the same in this case (and invariant to assignment probabilities), even though the data generation is itself not linear. The diagnosis reveals that the linear estimator is able to identify this linear estimand, despite being defined over variables that are non-linearly related to one another. However, as the unequal probability design shows, even with random assignment of Z to Y, the linear estimator can be biased. The diagnosis shows that variation in the assignment of different conditions also matters for inference, in addition to variation in assignment of units.

3.2.5 Matching on Observables

- *M Model*: We posit a population that has three standard normally distributed variables, X_1, X_2 and X_3. The potential outcomes of units in the population are an additive function of these variables and the treatment.
- *I Inquiry*: We wish to know the average effect of the treatment among those who were actually treated in a given implementation of the design.
- *D Data Strategy*: Units are assigned to treatment through a probit process that is a function of the X variables.
- *A Answer Strategy*: We match the units to one another using the three X variables and estimate the difference between treated and control among the matches.

3.2.5.1 Declaration

```
library(Matching)
# Model -----------------------------------------------------------------
population <- declare_population(
  N = 1000,
  X1 = rnorm(N),
  X2 = rnorm(N),
  X3 = rnorm(N))

# potential outcomes are correlated with the covariates
potential_outcomes <-
  declare_potential_outcomes(Y ~ X1 + X2 + X3 + Z)

# Data Strategy ---------------------------------------------------------
# treatment assignment is also correlated with the covariates
assignment <-
  declare_assignment(
    handler = function(data) {
      prob <- with(data, pnorm(X1 + X2 + X3))
      data$Z <- rbinom(nrow(data), 1, prob)
      return(data)})

reveal_Y <- declare_reveal(Y, Z)

# Inquiry ---------------------------------------------------------------
# The inquiry is defined *after* the data strategy, because it is the
# effect among those who happen to be treated
estimand <- declare_estimand(att = mean(Y_Z_1[Z == 1] - Y_Z_0[Z == 1]))

# Answer Strategy -------------------------------------------------------
# Estimator 1: DIM
estimator_d_i_m <- declare_estimator(Y ~ Z, estimand = "att", label = "dim")

# This helper returns the estimate from Matching::Match() in a data.frame
match_est <-
  function(data) {
    match_out <- with(data, Match(
      Y = Y,
      Tr = Z,
      X = cbind(X1, X2, X3)
    ))
    return(data.frame(term = "Z", estimate = match_out$est))
  }

# Estimator 2: Matching
estimator_m <- declare_estimator(
```

```
  handler = tidy_estimator(match_est),
  estimand = estimand,
  label = "matching")

matching <- population + potential_outcomes + assignment +
  reveal_Y + estimand + estimator_d_i_m + estimator_m
```

3.2.5.2 Diagnosis

```
if(do_diagnosis){
  diagnosis <-
    diagnose_design(
      matching,
      diagnosands = declare_diagnosands(select = bias),
      sims = sims, bootstrap_sims = b_sims)
}
```

Estimand Label	Estimator Label	Bias
att	dim	2.39<br>(0.00)
att	matching	0.52<br>(0.00)

The diagnosis reveals that matching provides a considerable improvement over the naive difference-in-means estimator in terms of bias with respect to the ATT. Nevertheless, even under these ideal conditions the matching estimator fails to provide completely unbiased estimates.

3.2.6 Regression Discontinuity

- *M Model*: We posit two potential outcomes functions, one for the treatment condition and another for the control. These functions are fourth-order polynomial equations that map the running variable X, to the outcome, Y. We suppose that X is drawn from a uniform distribution, and that units experience an idiosyncratic, normally distributed shock. The treatment variable is 1 when the running variable is greater than 0.5 (the cutoff) and 0 otherwise.
- *I Inquiry*: We wish to know the true difference in the potential outcomes functions at exactly the point on the running variable where the cutoff is located.
- *D Data Strategy*: We observe the available data without intervening or sampling.
- *A Answer Strategy*: Our estimator is a fourth order polynomial regression in which the terms are fully interacted with the treatment variable.

3.2.6.1 Declaration

```
cutoff <- 0.5

# Two "wiggly" potential outcome functions
control <- function(X) {
  as.vector(poly(X, 4, raw = TRUE) %*% c(0.7, -0.8, 0.5, 1))
  }

treatment <- function(X) {
  as.vector(poly(X, 4, raw = TRUE) %*% c(0, -1.5, 0.5, 0.8)) + 0.15
  }


rdd <-
  # Model -------------------------------------------------------------------
  declare_population(
    N = 1000,
    X = runif(N, 0, 1) - cutoff,
    noise = rnorm(N, 0, .1),
    Z = 1 * (X > 0)) +

  declare_potential_outcomes(Y_Z_0 = control(X) + noise,
                             Y_Z_1 = treatment(X) + noise) +
  # Inquiry -----------------------------------------------------------------
  # This is the difference in the PO functions *exactly* at the cutoff
  declare_estimand(LATE = treatment(0) - control(0)) +

  # Data Strategy -----------------------------------------------------------
  # Implicitly, we're sampling all of the data that is revealed
  # by which side of the cutoff a unit fall.
  declare_reveal(Y, Z) +

  # Answer Strategy ---------------------------------------------------------
  # here we're using an OLS model with 4th order polynomials on either side
  # of the cutoff
  declare_estimator(Y ~ poly(X, 4) * Z,
                    term = "Z",
                    model = lm_robust,
                    estimand = "LATE")
```

3.2.6.2 Diagnosis

```
if(do_diagnosis){
  diagnosis <-
    diagnose_design(
      rdd,
      sims = sims, bootstrap_sims = b_sims)
}
```

Estimand Label	Bias	RMSE	Power	Coverage
LATE	-0.06<br>(0.01)	0.89<br>(0.01)	0.05<br>(0.00)	0.95<br>(0.00)

3.2.7 Experimental Design

- *M Model*: Our model posits that all subjects are endowed with four potential outcomes, depending on whether they receive one, the other, both, or neither of two treatments. We allow the effects of each treatment to be different for each individual subject.
- *I Inquiry*: We wish to know the average effect of treatment 1 compared to the outcome when both treatments are absent.
- *D Data Strategy*: We compare two assignment strategies: a three-arm trial and a 2x2 factorial. In the three-arm trial, subjects can be exposed to either treatment or neither, but not both treatments together. By contrast, in the 2x2 factorial, all four combinations are possible.
- *A Answer Strategy*: We regress the outcome on indicators for both treatment conditions. In addition, we examine the interaction between treatments in the 2x2 factorial case only.

3.2.7.1 Declaration

Three-Arm Design

We here use the `multi_arm_designer()` function from the `DesignLibrary` to quickly declare a design that has an N of 500, and three treatment conditions assigned with equal probability, with each non-control treatment generating an effect of 0.2.

```
three_arm_design <-
  multi_arm_designer(N = 500,
                     m_arms = 3,
                     outcome_means = c(0, 0.2, 0.2))
```

2x2 Factorial Design

We similarly use the `two_by_two_designer()` function to quickly declare a design that has an N of 500, and four treatment conditions assigned with equal probability. Again, each non-control treatment generates an effect of 0.2 when the other treatment is absent. Their interaction produces an additional interactive effect of 0.2.

```
two_by_two_design <-
  two_by_two_designer(N = 500,
                      outcome_means = c(0, 0.2, 0.2, 0.6))
```

We are interested in comparing the two approaches as the size of the interaction increases. We consider nine values of the interaction between -0.2 and 0.2.

```
interactions <- seq(-0.2, 0.2, length.out = 9)
means <- lapply(interactions, function(x) c(0, 0.2, 0.2, 0.4 + x))

two_by_two_designs <-
  expand_design(
    designer = two_by_two_designer,
    prefix = "factorial",
    N = 500,
    weight_A = 0,
    weight_B = 0,
    outcome_means = means
  )
```

3.2.7.2 Diagnosis

```
if(do_diagnosis){
  diagnosis_1 <-
    diagnose_design(
```

```
      three_arm_design,
      sims = sims, bootstrap_sims = b_sims)

  diagnosis_2 <-
    diagnose_design(
      two_by_two_designs,
      sims = sims, bootstrap_sims = b_sims)
}
```

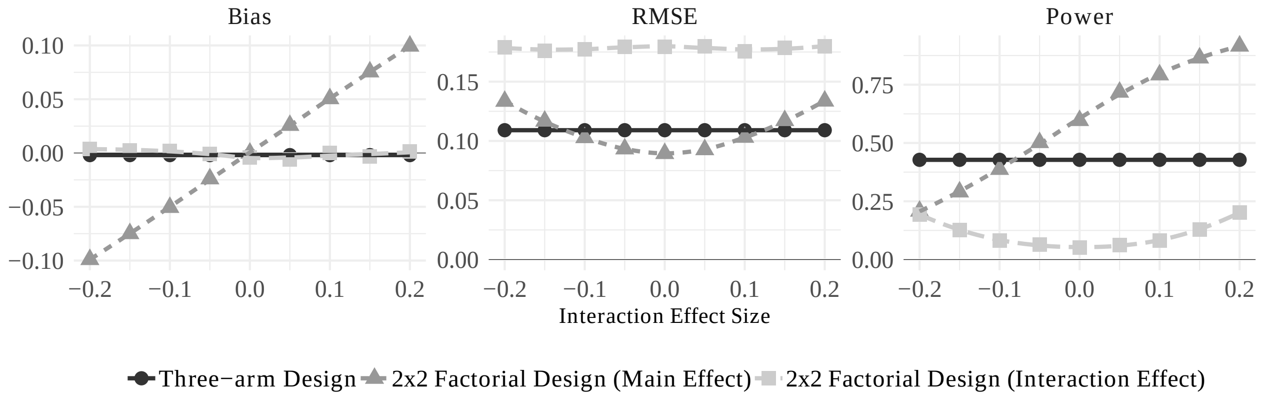

The diagnosis reveals that neither design exhibits bias when the true interaction term is equal to zero. However, as the interaction between the two treatments is stronger, the factorial design renders estimates of the effect of treatment that are more and more biased relative to the 'pure' main effect estimand. Moreover, there is a bias-variance tradeoff in choosing between the two designs when the interaction is weak. When the interaction term is close to zero, the factorial design is preferred, because it is more powerful: it compares one half of the subject pool to the other half, whereas the three arm design only compares a third to a third. However, as the magnitude of the interaction term increases, the precision gains are offset by the increase in bias.

3.3 Designs for Discovery Research

- *M Model*: The population consists of two groups (men and women, for instance). The conditional average treatment effect is sometimes larger in one group than another.
- *I Inquiry*: The main purpose of the experiment is to estimate the overall average treatment effect (ATE). Depending on whether significantly different subgroup are found are found, the difference in subgroup effects becomes a second question of interest, resulting from discovery.
- *D Data Strategy*: We allocate treatment using complete random assignment.
- *A Answer Strategy*: Using a random half of the data (the training set), we test for an interaction of treatment with group membership, i.e. the difference in conditional average treatment effects (CATE). If we find a significant interaction at the $p \leq 0.05$ level, we declare the interaction as a new estimand and estimate the size of the interaction in the test dataset.

We also include, for comparison, an analysis that reports the interaction estimated using the full data **whenever that interaction is significant**.

3.3.0.1 Declaration

```
# Model ---------------------------------------------------------------
population <-
  declare_population(
    N = 200,
    X = draw_binary(prob = rep(0.5, N)),
    heterogenous_effect = sample(c(0, .5), 1, TRUE),
    train = draw_binary(prob = rep(0.5, N)),
    u = rnorm(N))

pos <- declare_potential_outcomes(Y ~ Z + heterogenous_effect * Z * X + u)

# Inquiry -------------------------------------------------------------

estimand <- declare_estimand(ATE = mean(Y_Z_1 - Y_Z_0))

# Data Strategy -------------------------------------------------------
assignment <- declare_assignment(prob = 0.5)
reveal <- declare_reveal(Y, Z)

# Answer Strategy -----------------------------------------------------

# here we do the main analysis (estimate the ATE)
main_analysis <-
  declare_estimator(Y ~ Z, estimand = "ATE", label = "Main")

# Here we start exploring
explore <-
  declare_step(
    train_pval = tidy(lm_robust(Y ~ Z * X, subset = train == 1))[4, 4],
    all_pval = tidy(lm_robust(Y ~ Z * X))[4, 4],
    handler = fabricate)

# We write down a second inquiry in the "discovery" section
# to indicate that we possibly "discover" that we are interested
# in this question, the difference-in-CATEs for X = 1 vs X = 0
discovery_estimand <-
  declare_estimand(
    diff_in_CATEs = mean(Y_Z_1[X == 1] - Y_Z_0[X == 1]) -
                    mean(Y_Z_1[X == 0] - Y_Z_0[X == 0]))
```

```
# This estimator is *conditional* on significance in the training set
test_sample_estimator <-
  function(data){
    if(data$train_pval[1] < 0.05) {
      fit <- lm_robust(Y ~ Z*X, subset = train == 0, data = data)
      return(tidy(fit)[4, c("estimate", "p.value", "term")])
    } else {
      return(data.frame(estimate = NA, p.value = NA, term = "Z:X", stringsAsFactors = FALSE))
    }
  }

test_sample_analysis <-
  declare_estimator(
    handler = tidy_estimator(test_sample_estimator),
    estimand = discovery_estimand,
    label = "Test sample")

# This estimator is *conditional* on significance in the full data
full_sample_estimator <-
  function(data){
    if(data$all_pval[1] < 0.05) {
      fit <- lm_robust(Y ~ Z*X, data = data)
      return(tidy(fit)[4, c("estimate", "p.value", "term")])
    } else {
      return(data.frame(estimate = NA, p.value = NA, term = "Z:X", stringsAsFactors = FALSE))
    }
  }

full_sample_analysis <-
  declare_estimator(
    handler = tidy_estimator(full_sample_estimator),
    estimand = discovery_estimand,
    label = "Full sample")

discovery <- population + pos + estimand + assignment + reveal +
  main_analysis + explore + discovery_estimand + test_sample_analysis +
  full_sample_analysis

discovery_diagnosands <-
  declare_diagnosands(
    bias = mean((estimate - estimand), na.rm = TRUE),
    RMSE = sqrt(mean((estimate - estimand)^2, na.rm = TRUE)),
    frequency = mean(!is.na(estimate)),
    false_pos = mean(p.value[estimand == 0] < 0.05, na.rm = TRUE),
    false_neg = 1 - mean(p.value[estimand != 0] < 0.05, na.rm = TRUE),
    keep_defaults = FALSE)
```

3.3.0.2 Diagnosis

```
if(do_diagnosis){
  diagnosis <-
    diagnose_design(
      discovery,
      diagnosands = discovery_diagnosands,
      sims = sims, bootstrap_sims = b_sims)
}
```

Estimator Label	Term	Bias	RMSE	Frequency	False Pos	False Neg
Main	Z	0.00	0.14	1.00	NaN	0.00
		(0.00)	(0.00)	(0.00)	NA	(0.00)
Full sample	Z:X	-0.25	0.32	0.29	0.05	1.00

Estimator Label	Term	Bias	RMSE	Frequency	False Pos	False Neg
		(0.01)	(0.01)	(0.01)	(0.01)	(0.00)
Test sample	Z:X	-0.00	0.41	0.32	0.05	0.78
		(0.01)	(0.01)	(0.01)	(0.01)	(0.02)

We see that the principled discovery method, using training and testing data, provides essentially unbiased estimates of the heterogeneous effect, whereas the comparison method tends to provide biased estimates, because conditioning on statistical significance tends to exaggerate effect sizes (Gelman and Carlin 2014).

The consequences of the two approaches for false discovery rates are stark. If the true effect is 0, the probability of falsely rejecting the null of 0 (conditional on reporting) is 1 under the comparison method: by definition, only significant estimates are kept. Similarly, since the only estimates generated by this procedure are statistically significant, the false negative rate (conditional on reporting) is 0. This is because the estimand is declared and the analysis is conducted only when estimates are guaranteed to be significant. The principled discovery method exhibits conventional rates for falsely rejecting a true null (0.05) though it it fails to reject the null quite often, due to weak power.

The protection from bias from the principled discovery strategy declared here does not necessarily translate into improved inferences on average, because of a bias-variance tradeoff inherent in the approach. A smaller sample is used in the final test when adopting a principled discovery approach, and so on average the estimates are much noisier than under the unprincipled comparison.

Moreover, the principled strategy is somewhat less likey to produce a result at all since it is less likely that a result would be discovered in a subset of the data than in the entire data set.

4 Bjorkman and Svensson (2009) Design Replication

We present a “design replication” of Björkman and Svensson (2009), by which we mean an exercise in which we learn about the design of a study that has already been conducted. Note that a design replication requires making assumptions about expected features of the data generation processes as well as treatment effects; researchers can disagree on these features. The design replication provides information on features of the design conditional on these assumptions. This exercise is intended to demonstrate how careful specification of estimands can shed light on – and quantify – otherwise hard to assess limitations of analytic strategies.

The study reports the results of a cluster-randomized trial of the effects of community-based monitoring of health clinics in Uganda. The unit of assignment is the health clinic but measurement takes place at the level of the household. Households are considered treated if they are located within the catchment area (5km radius) of a treated health clinic.

The experiment focuses on improvements in two main health outcomes: reductions in child mortality and increases in child weight. The first outcome is measured as the catchment-area-level under-5 mortality rate, expressed in death rates per 1000 live births. In the control group, this rate was 144, compared with 97 in the treatment group: a 33% reduction in child mortality. The second outcome (measured at the household level) is the weight-for-age of infants, defined as children under 18 months. Weight-for-age is measured in standard units, so the positive 0.14 coefficient estimate implies that the weight-for-age of infants in the treatment group was 0.14 standard deviations higher.

We will now characterize this design using the MIDA framework.

4.1 Model

The population of interest comprises the households within the catchment areas of the 50 health clinics. When we declare the population, we will create three background covariates, two at the household level and one at the catchment area level.

1. `infant`: indicator that equals one if an infant was born into a household in the 18 months preceding the treatment. This variable is observable.
2. `family_health`: a normally distributed variable that represents the health of the household. This variable is likely to be unobservable. We cannot measure it, but it will be positively correlated with the `weight_for_age` of surviving children.
3. `area_health`: a normally distributed variable that represents the overall health of the community. This variable will be the same for all households living within a catchment area and will ensure that outcomes are correlated within catchment area. This variable is also unobservable.

The data are hierarchical – there are 2500 households in each of 50 clusters. The resulting 125,000 row dataset is the population from which subjects will be sampled.

```
# Number of clusters in original study
N_catchment_areas <- 50

# Estimated probability of having a child
infant_prob <- (1135 / (1 - 0.1205)) / 5000

pop <- declare_population(
  catchment_area = add_level(
    N = 300, area_health = rnorm(N)),
  households = add_level(
    N = 2500, infant = rbinom(n = N, 1, prob = infant_prob),
    family_health = rnorm(N)))

fixed_pop <- declare_population(data = pop())
```

The two outcomes of interest are infant mortality and infant weight. We will first build the infant mortality potential outcomes with a custom function. This custom function builds the probability of an infant surviving in terms of a logistic model, then draws from a binomial distribution using the resulting probabilities. We assume that there is a base rate of survival of approximately 86%, and that treatment increases the probability of survival by approximately 5 percentage points. In logits, this is moving from `plogis(1.81) = 86\%` to `plogis(1.81 + 0.5) = 91\%`. The probability of survival is also positively correlated with the latent health of the household and the health of the community. Finally, if a household does not have an infant, then this potential outcome is undefined. We denote treatment status as $Z = 0$ for control and $Z = 1$ for treatment, hence the condition labels `Z0` and `Z1`.

```
alive_po_function <- function(Z, family_health, area_health, infant) {
  alive <- rbinom(
    n = length(Z), size = 1,
    prob = plogis(qlogis(0.86) + 0.5 * Z + family_health + area_health))
  alive[infant == 0] <- NA
  return(alive)}

pos_alive <- declare_potential_outcomes(
  infant_alive ~ alive_po_function(Z, family_health, area_health, infant))
```

The second potential outcome is the `weight\_for\_age` of surviving infants. This potential outcome is equal to the latent health of the household for control units. The treated potential outcome is the sum of the latent health and the 0.14 standard deviation treatment effect. Finally, this outcome is masked if the infant dies or if the household does not have an infant.

```
weight_po_function <-
  function(Z, infant_alive_Z_0, infant_alive_Z_1, family_health, area_health) {
    weight <- 0.14 * Z + family_health + area_health
    masked <- infant_alive_Z_1 * Z + infant_alive_Z_0 * (1 - Z)
    weight[(masked) == 0 | is.na(masked)] <- NA
    return(weight)}

pos_weight <- declare_potential_outcomes(
  weight_for_age ~ weight_po_function(
    Z, infant_alive_Z_0, infant_alive_Z_1, family_health, area_health))
```

4.2 Inquiry

We have two inquiries, the average effect on child mortality (at the cluster level) and the average effect on weight-for-age at the household level.

```
cl_mortality_estimand <- declare_estimand(
  Mortality = (1 - mean(infant_alive_Z_1)) -
    (1 - mean(infant_alive_Z_0))
)

hh_weight_estimand <- declare_estimand(
  Weight = mean(weight_for_age_Z_1 - weight_for_age_Z_0),
  subset = infant == TRUE & infant_alive_Z_0 == 1 & infant_alive_Z_1 == 1
)
```

The second estimand has a complication – it is only defined for a subset of the population. The table below shows four types of infants: Type A (for “Adverse”) is alive if in control, but dies if in treatment. Type B (“Beneficial”) is just the reverse: the child dies if untreated, but survives if treated. Type C (“Chronic”) would die under either condition and Type D (“Destined”) would live under either condition. For the first three types, the child dies under one condition, the other or both. This means that the difference in weight potential outcomes is undefined for those types. The difference in weight due to treatment is only defined for Type D infants, those who would survive under either treatment. We therefore define the estimand as being the difference in outcomes for Type D.

This estimand is not recoverable from this design, as we cannot distinguish type A from type D in the control group and type B from type D in the treatment group.

Type	Alive $(Z = 0)$	Alive $(Z = 1)$	Weight $(Z = 0)$	Weight $(Z = 1)$	Estimand
A	1	0	exists	NA	undefined
B	0	1	NA	exists	undefined
C	0	0	NA	NA	undefined
D	1	1	exists	exists	E[Weight(Z=1) - Weight(Z=0)]

```
hh_weight_estimand <- declare_estimand(
  Weight = mean(weight_for_age_Z_1 - weight_for_age_Z_0),
  subset = infant == 1 & infant_alive_Z_0 == 1 & infant_alive_Z_1 == 1
)
```

4.3 Data Strategy

Our data strategy includes both the stratified sampling of households by catchment areas and the random assignment of catchment areas to treatment or control. Since the target is 5,000 total households, the study samples 100 households from each catchment area. Assignment to treatment is straightforward: 25 of the 50 clusters receive treatment.

```
cl_sampling <- declare_sampling(clusters = catchment_area, n = N_catchment_areas)
hh_sampling <- declare_sampling(strata = catchment_area, prob = 100/2500)

assignment <- declare_assignment(clusters = catchment_area)

reveal_outcomes <- declare_reveal(outcome_variables = c(infant_alive, weight_for_age))
```

4.4 Answer Strategy

The two estimands require different estimation procedures. For the mortality estimand, we first aggregate the data up to the cluster level, then take the difference in cluster means.

```
aggregate_data <- declare_step(
  handler = function(data){
    aggregate(cbind(infant_alive_Z_0, infant_alive_Z_1, infant_alive, Z) ~ catchment_area,
              FUN = mean, na.rm = TRUE, data = data)
  })

cl_mortality_estimator <- declare_estimator(
  (1 - infant_alive) ~ Z,
  model = lm_robust,
  estimand = cl_mortality_estimand,
  label = "Mortality estimate")
```

The second estimand is at the household level, but we must nevertheless cluster our standard errors by the catchment area. Note that we estimate this quantity among all observed values of `weight\_for\_age`. In the control group, the observed values are a mixed of types A and D, and in the treatment group, the values are a mixture of types B and D. Ideally, we would subset the estimation to include only Type D households, but this information requires knowledge of both the treated and untreated potential outcomes, which is impossible. If potential outcomes are correlated with type (as they are in this simulation), this estimator is biased.

```
hh_weight_estimator <- declare_estimator(
  weight_for_age ~ Z,
  model = lm_robust,
  clusters = catchment_area,
  estimand = hh_weight_estimand,
  label = "Weight estimate")
```

4.5 Diagnosis of original design

We now provide the `diagnose_design()` function with the declarations we made above. We will draw a large finite population once, then for each simulation, draw a stratified sample, allocate treatments, reveal outcomes, and conduct the estimation.

```
bjorkman_svensson_design <-
  fixed_pop +
  pos_alive +
  pos_weight +
  cl_sampling +
  hh_sampling +
  assignment +
  reveal_outcomes +
  hh_weight_estimator +
  hh_weight_estimand +
  aggregate_data +
  cl_mortality_estimand +
  cl_mortality_estimator
```

```
if(do_diagnosis){
  diagnosis_1 <-
    diagnose_design(
      bjorkman_svensson_design,
      sims = sims, bootstrap_sims = b_sims)
}
```

Estimand Label	Mean Estimand	Mean Estimate	Bias	Power
Mortality	-0.06	-0.06	0.00	0.29
	(0.00)	(0.00)	(0.00)	(0.01)
Weight	0.14	0.06	-0.08	0.06
	(0.00)	(0.00)	(0.00)	(0.00)

The summary of the diagnosis output is presented in the table above. Considering the under-5 mortality rate first, we see that the true population average treatment effect is 0.006 percentage points. In our simulations, we estimate the true standard error to be 0, which is close to the standard error reported in the original paper of 0.026. The coverage is correct, at 95%. The simulation presented above shows that we are relatively under-powered for the mortality estimand, only 0.1% of simulations returned a statistically significant result.

Turning next to the weight-for-age analysis, the simulations reveal that our estimator is biased. Because we built into our potential outcomes the assumption that less-health infants were the ones who are most likely to be of type B ("Beneficial"), the treatment group mean is pulled down. Under this assumption, the bias is downwards – our analysis systematically understates the effect on weight-for-age among type D infants, the only type for whom the estimand is defined.

```
summary_df <-
  diagnosis_1 %>%
  get_simulations() %>%
  gather(key, value, estimate, estimand) %>%
  group_by(estimand_label, key) %>%
  summarize(est = mean(value)) %>%
  mutate(label = paste0("Mean ", key))

g <-
  diagnosis_1 %>%
  get_simulations() %>%
  group_by(estimand_label) %>%
  mutate(mean_estimand = mean(estimand)) %>%
  ggplot(aes(estimate)) +
  geom_histogram(bins = 50,alpha = .5) +
```

```
  geom_vline(xintercept = 0, color = "black", size = .3) +
  geom_vline(data = summary_df, aes(xintercept = est, linetype = label), size = 0.8) +
  scale_linetype_manual(name = "",values = c("dotted","dashed")) +
  facet_wrap(~estimand_label, scales="free_x") +
  ylab('Frequency') +
  xlab("Sampling Distributions of the Two Estimators") +
  theme_bw() +
  theme(
    axis.ticks = element_blank(),
    axis.line = element_blank(),
    panel.border = element_blank(),
    panel.grid.major = element_line(color = '#eeeeee'),
    strip.background = element_blank(),
    legend.position = "bottom",
    text = element_text(family = "Palatino"),
    axis.text = element_text(size = 12),
    strip.text = element_text(size = 12))
```

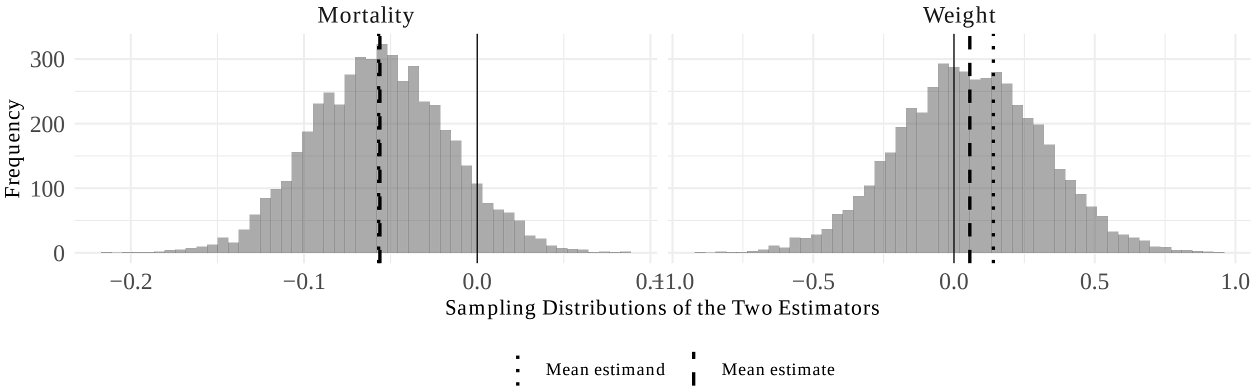

4.6 Increasing Sample Size

The preceding diagnosis suggests that the original design exhibits low power given the posited model of the data-generating process. Gelman and Carlin (2014) has highlighted how low power can generate bias (even in experiments) if researchers and critics restrict their inferences about the underlying effect to statistically significant estimates. Such bias arises from the so-called "statistical significance filter": only abnormally large effect estimates will be significant when power is low.

To measure the risk of bias arising from consumers applying a "statistical significance filter," we can examine what Gelman and Carlin (2014) refer to as the "exaggeration ratio." The "exaggeration ratio" tells us the expected absolute value of the estimate relative to the absolute value of the estimand, given that the estimate is statistically significant at some level.

We here examine the exggeration ratio, and in particular compare it to the exaggeration ration one might expect from the replication exercise conducted by Raffler, Posner, and Parkerson (2019). Specifically, we analyze the extent of the design improvement that results from the increased number of clusters, given that all of our other assumptions about the original design remain unchanged.

We must first decide how much to augment the sample size. Raffler, Posner, and Parkerson (2019) split the original Power to the People intervention into what they determine to be its two most important components: the provision of information and mobilization of health teams and members of the community, on the one hand, and the implementation of meetings for community members and health staff to plan and raise issues, on the other. They use a factorial design, in which 95 clusters are assigned to control, 97 to meetings without information, 92 to information without meetings, and 92 to a combination of information and meetings. Thus, while the RPP replication augments the total sample size to 376, compared to 50 clusters in the original, if

we compare clusters assigned to directly comparable treatment arms we have the 95 assigned to control and the 92 assigned to the full combination.

We keep the same design as before, but increase the number of clusters to 187.

```
RPP_replication <- redesign(bjorkman_svensson_design, N_catchment_areas = (95 + 92))
```

We diagnose the two designs, declaring the new "exaggeration ratio" diagnosand and setting $\alpha = 0.1$.

```
if(do_diagnosis) {
  diagnosis_2 <-
    diagnose_design(
      bjorkman_svensson_design,
      RPP_replication,
      diagnosands = declare_diagnosands(
        exaggeration_ratio = mean(abs(estimate[p.value < 0.1]) / abs(estimand[p.value < 0.1])),
        select = "power"
      ),
      sims = sims,
      bootstrap_sims = b_sims
    )
}
```

The diagnoses reveal a substantial improvement but highlight ongoing concerns about statistical significance filters. According to this exercise, the original study risked exaggerating the size of the weight effect by a factor of 4, and the mortality effect by a factor of almost 2. By contrast, increasing the number of clusters as in Raffler, Posner, and Parkerson (2019) improves power and so all but eliminates the risk that statistically significant estimates exaggerate the true underlying effect on mortality. Nevertheless, even with the substantial increase in power, the weight estimate is still at risk of exaggeration, in part due to the bias.

Design Label	N_catchment_areas	Estimator Label	Exaggeration Ratio	Power
bjorkman_svensson_design	50	Mortality estimate	1.58 (0.01)	0.28 (0.01)
bjorkman_svensson_design	50	Weight estimate	3.73 (0.03)	0.05 (0.00)
RPP_replication	187	Mortality estimate	1.08 (0.00)	0.80 (0.01)
RPP_replication	187	Weight estimate	2.02 (0.02)	0.07 (0.00)

4.7 Adding Covariates

In their analytic replication of Björkman and Svensson (2009), Donato and Garcia Mosqueira (2016) (D&M) note that the eighteen community-based organizations (CBOs) who carried out the original "Power to the People" intervention were active in 64 percent of the treatment communities and 48 percent of the control communities. The replicators posit that the presence of CBOs may be correlated with health outcomes, and therefore include in their analytic replication of the mortality and weight-for-age regressions both an indicator for CBO presence and the interaction of the intervention with CBO presence.

The original authors (B&S) criticized the replicators' decision to include CBO presence as a regressor, on the grounds that in any such study it is possible to find some unrelated variable whose inclusion will increase standard errors or decrease the coefficient of interest.

Expressed in terms of MIDA, we have two conflicting claims about the Model: B&S claim that CBO presence is unrelated to the outcome of interest, whereas D&M claim that CBO presence might indeed affect health outcomes. Moreover, we have the proposal of different answer strategies, with D&M claiming that an indicator for CBO presence and even an interaction of the main treatment indicator with CBO presence should be included in the estimator. How can we assess the grounds for these competing claims?

Since we do not know whether the replicators would have conditioned on CBO presence and its interaction with the intervention if it had not been imbalanced, we modify the original design to include four different estimation strategies: the first ignores CBO presence as in the original study; the second includes CBO presence irrespective of imbalance; the third includes an indicator for CBO presence only if the CBO presence is "significantly" imbalanced among the 50 treatment and control clusters (at the $\alpha = .05$ level); and the last strategy includes terms for both CBO presence and an interaction of CBO presence with the treatment irrespective of imbalance.

```
conditional_estimator <- function(data, strategy, alpha = 0.1){
  imbalanced_fit <- lm_robust(formula = CBO ~ Z,data = data,clusters = catchment_area)
  imbalanced <- imbalanced_fit$p.value["Z"] < alpha
  how_imbalanced <- imbalanced_fit$coefficients["Z"] /
                    imbalanced_fit$coefficients["(Intercept)"]
  if(strategy == "ignore") formula <- as.formula(weight_for_age ~ Z)
  if(strategy == "include") formula <- as.formula(weight_for_age ~ Z + CBO)
  if(strategy == "interact") formula <- as.formula(weight_for_age ~ Z + CBO + Z : CBO)
  if(strategy == "include if imbalanced") {
    if(imbalanced) formula <- as.formula(weight_for_age ~ Z + CBO)
    else formula <- as.formula(weight_for_age ~ Z)
  }
  cbind(tidy(lm_robust(formula = formula, data = data, clusters = catchment_area))[2,],
        imbalanced = imbalanced, how_imbalanced = how_imbalanced)
}
ignore_CBO <- declare_estimator(
  handler = tidy_estimator(conditional_estimator),
  strategy = "ignore",
  estimand = hh_weight_estimand,
  label = "Ignore CBO")
include_CBO <- declare_estimator(
  handler = tidy_estimator(conditional_estimator),
  strategy = "include",
  estimand = hh_weight_estimand,
  label = "Include CBO")
interact_CBO <- declare_estimator(
  handler = tidy_estimator(conditional_estimator),
  strategy = "interact",
  estimand = hh_weight_estimand,
  label = "Interact CBO")
include_CBO_if_imbalanced <- declare_estimator(
  handler = tidy_estimator(conditional_estimator),
  strategy = "include if imbalanced",
  estimand = hh_weight_estimand,
  label = "Include CBO if imbalanced")
```

We consider how these strategies perform under a model in which, as claimed by the authors, CBO presence is unrelated to health outcomes, and another in which, as claimed by the replicators, CBO presence is highly correlated with health outcomes.

We firstly specify that the correlation is 0 and add a random draw of CBOs to the catchment area level, and redeclare the design. This is consistent with the claim made by B&S, namely that CBO presence is not correlated with health outcomes.

```
CBO_correlation <- 0

fixed_pop_CBO <- declare_population(
  data = pop(),
  catchment_area = modify_level(
    CBO = correlate(draw_handler = draw_binary,
                    given = area_health,
                    rho = CBO_correlation,
                    prob = (.64 + .48) / 2)
  )
```

```
)

DM_replication_CBO_ind <-
  fixed_pop_CBO +
  pos_alive +
  pos_weight +
  cl_sampling +
  hh_sampling +
  assignment +
  reveal_outcomes +
  hh_weight_estimand +
  ignore_CBO +
  include_CBO +
  interact_CBO +
  include_CBO_if_imbalanced
```

We then declare an alternative design in which CBO presence is highly correlated with health outcomes in the catchment area.

```
DM_replication_CBO_cor <- redesign(design = DM_replication_CBO_ind, CBO_correlation = .8)
```

And we here assess how well the various answer strategies proposed by the authors fare under the differing assumptions. Importantly, we look both at the overall mean-squared error, and at the mean-squared error when CBO presence is and is not significantly imbalanced at the cluster level. These conditional diagnosands shed light on the consequences of only including a covariate when it is imbalanced.

```
if(do_diagnosis){
  diagnosis_3 <-
    diagnose_design(
      DM_replication_CBO_ind, DM_replication_CBO_cor,
      diagnosands = declare_diagnosands(
        rmse_balanced = sqrt(mean((estimate[!imbalanced] - estimand[!imbalanced])^2)),
        rmse_imbalanced = sqrt(mean((estimate[imbalanced] - estimand[imbalanced])^2)),
        select = "rmse"),
      sims = sims, bootstrap_sims = b_sims)
}
```

We note first that including the interaction term as done by the replicators is a strictly dominated strategy from the standpoint of reducing mean squared error: irrespective of whether CBO presence is correlated with health outcomes or imbalanced, the RMSE expected under this strategy is higher than under any other strategy. This might not be the case for a strategy that first demeaned CBO presence before interacting so that the coefficient on treatment shoots at the average effect rather than the effect when CBO = 0. Thus, based on a criterion of "Homeground Dominance" in favor of B&S, one would be justified in discounting the importance of the replicators' observation that the interaction diminishes the significance of the main effect.

Supposing now that there is no correlation between CBO presence and health outcomes, inclusion of the CBO indicator does increase RMSE ever so slightly in those instances where there is imbalance and the standard errors are ever so slightly larger. On average, however, the strategies of conditioning on CBO presence regardless of balance and conditioning on CBO presence only if imbalanced perform about as well as a strategy of ignoring CBO presence when there is no underlying correlation. However, when there is correlation in health outcomes and CBO presence, strategies that include CBO presence improve RMSE considerably, especially when there is imbalance. Thus, D&M could make a "Robustness to Alternative Models" claim in defense of their strategy: including CBO presence does not greatly diminish inferential quality if you do not agree with their claim about the model, and improves it if you do.

CBO_correlation	Estimator Label	RMSE	RMSE Balanced	RMSE Imbalanced
0	Ignore CBO	0.27<br>(0.00)	0.27<br>(0.00)	0.26<br>(0.01)
0	Include CBO	0.27<br>(0.00)	0.27<br>(0.00)	0.26<br>(0.01)

CBO_correlation	Estimator Label	RMSE	RMSE Balanced	RMSE Imbalanced
0	Include CBO if imbalanced	0.27	0.27	0.26
		(0.00)	(0.00)	(0.01)
0	Interact CBO	0.28	0.28	0.27
		(0.00)	(0.00)	(0.01)
0.8	Ignore CBO	0.27	0.23	0.49
		(0.00)	(0.00)	(0.01)
0.8	Include CBO	0.19	0.17	0.31
		(0.00)	(0.00)	(0.01)
0.8	Include CBO if imbalanced	0.24	0.23	0.31
		(0.00)	(0.00)	(0.01)
0.8	Interact CBO	0.24	0.22	0.34
		(0.00)	(0.00)	(0.01)

References

Björkman, Martina, and Jakob Svensson. 2009. "Power to the People: Evidence from a Randomized Field Experiment of a Community-Based Monitoring Project in Uganda." *Quarterly Journal of Economics* 124 (2): 735–69.

Donato, Katherine, and Adrian Garcia Mosqueira. 2016. "Power to the People? A Replication Study of a Community-Based Monitoring Programme in Uganda." *3ie Replication Papers* 11. 3ie.

Duşa, Adrian. 2018. *QCA with R. A Comprehensive Resource.* New York: Springer.

Duşa, Adrian, and Alrik Thiem. 2015. "Enhancing the Minimization of Boolean and Multivalue Output Functions with E Qmc." *The Journal of Mathematical Sociology* 39 (2). Taylor & Francis: 92–108.

Fairfield, Tasha. 2013. "Going Where the Money Is: Strategies for Taxing Economic Elites in Unequal Democracies." *World Development* 47. Elsevier: 42–57.

Fairfield, Tasha, and Andrew E. Charman. 2017. "Explicit Bayesian Analysis for Process Tracing: Guidelines, Opportunities, and Caveats." *Political Analysis* 25 (3). Cambridge: Cambridge University Press: 363–80.

Gelman, Andrew, and John Carlin. 2014. "Beyond Power Calculations Assessing Type S (Sign) and Type M (Magnitude) Errors." *Perspectives on Psychological Science* 9 (6): 641–51.

Green, Peter, and Catriona J. MacLeod. 2016. "SIMR: An R Package for Power Analysis of Generalized Linear Mixed Models by Simulation." *Methods in Ecology and Evolution* 7 (4). Wiley Online Library: 493–98.

Groemping, Ulrike. 2016. "Design of Experiments (Doe) & Analysis of Experimental Data." https://cran.r-project.org/web/views/ExperimentalDesign.html.

Guo, Yi, Henrietta L. Logan, Deborah H. Glueck, and Keith E. Muller. 2013. "Selecting a Sample Size for Studies with Repeated Measures." *BMC Medical Research Methodology* 13 (1): 100. https://doi.org/10.1186/1471-2288-13-100.

Humphreys, Macartan, and Alan M. Jacobs. 2015. "Mixing Methods: A Bayesian Approach." *American Political Science Review* 109 (4). Cambridge: Cambridge University Press: 653–73.

Kreidler, Sarah M., Keith E. Muller, Gary K. Grunwald, Brandy M. Ringham, Zacchary T. Coker-Dukowitz, Uttara R. Sakhadeo, Anna E. Barón, and Deborah H. Glueck. 2013. "GLIMMPSE: Online Power Computation for Linear Models with and Without a Baseline Covariate." *Journal of Statistical Software* 54 (10). NIH Public Access.

Lieberman, Evan S. 2005. "Nested Analysis as a Mixed-Method Strategy for Comparative Research." *American Political Science Review* 99 (3). Cambridge: Cambridge University Press: 435–52.

Raffler, Pia, Daniel N. Posner, and Doug Parkerson. 2019. "The Weakness of Bottom-up Accountability: Experimental Evidence from the Ugandan Health Sector."

Ragin, Charles. 1987. *The Comparative Method. Moving Beyond Qualitative and Quantitative Strategies*. Berkeley: University of California Press.

Rohlfing, Ingo. 2018. "Power and False Negatives in Qualitative Comparative Analysis: Foundations, Simulation and Estimation for Empirical Studies." *Political Analysis* 26 (1). Cambridge: Cambridge University Press: 72–89.

Thiem, Alrik, and Adrian Dusa. 2013. "QCA: A Package for Qualitative Comparative Analysis." *The R Journal* 5 (1): 87–97.
